# Reconstitution of the immune system and clinical correlates after stem cell transplantation for systemic sclerosis

**DOI:** 10.3389/fimmu.2022.941011

**Published:** 2022-08-11

**Authors:** Marianna Y. Kawashima-Vasconcelos, Maynara Santana-Gonçalves, Djúlio C. Zanin-Silva, Kelen C. R. Malmegrim, Maria Carolina Oliveira

**Affiliations:** ^1^ Center for Cell-Based Therapy, Regional Hemotherapy Center of the Ribeirão Preto Medical School, University of São Paulo, Ribeirão Preto, Brazil; ^2^ Internal Medicine Graduate Program, Ribeirão Preto Medical School, University of São Paulo, Ribeirão Preto, Brazil; ^3^ Oncology, Stem Cell and Cell-Therapy Graduate Program, Ribeirão Preto Medical School, University of São Paulo, Ribeirão Preto, Brazil; ^4^ Basic and Applied Immunology Graduate Program, Ribeirão Preto Medical School, University of São Paulo, Ribeirão Preto, Brazil; ^5^ Department of Clinical, Toxicological and Bromatological Analysis, School of Pharmaceutical Sciences of Ribeirão Preto, University of São Paulo, Ribeirão Preto, Brazil; ^6^ Department of Internal Medicine, Ribeirão Preto Medical School, University of São Paulo, Ribeirão Preto, Brazil

**Keywords:** systemic sclerosis, hematopoietic stem cell transplantation, immune reconstitution, immune monitoring, immune tolerance, cellular therapy

## Abstract

Systemic sclerosis (SSc) is a chronic autoimmune disease that includes fibrosis, diffuse vasculopathy, inflammation, and autoimmunity. Autologous hematopoietic stem cell transplantation (auto-HSCT) is considered for patients with severe and progressive SSc. In recent decades, knowledge about patient management and clinical outcomes after auto-HSCT has significantly improved. Mechanistic studies have contributed to increasing the comprehension of how profound and long-lasting are the modifications to the immune system induced by transplantation. This review revisits the immune monitoring studies after auto-HSCT for SSc patients and how they relate to clinical outcomes. This understanding is essential to further improve clinical applications of auto-HSCT and enhance patient outcomes.

## Introduction

Autologous hematopoietic stem cell transplantation (auto-HSCT) is considered for patients with severe and progressive autoimmune diseases. In the past 30 years, much has improved in the field, especially concerning patient care. Developments in patient selection, choice of conditioning regimens, and intra-transplant patient management have significantly decreased transplant-related toxicity and improved patient outcomes (1). Recently, auto-HSCT has been included in the recommendations for treating diseases such as systemic sclerosis and multiple sclerosis (2–4). For the remaining autoimmune diseases, transplants are mostly limited to clinical trials.

Auto-HSCT eliminates abnormal immune cells and reconstitutes a new, self-tolerant, long-lasting immunological system (1, 5). As a first phase of the procedure, autologous hematopoietic stem and progenitor cells are mobilized from the bone marrow to the peripheral blood, harvested by apheresis, and subsequently cryopreserved (1). Then, a conditioning regimen is administered to ablate the patient’s autoreactive immune system using chemotherapy or radiation agents associated with lymphocyte-depleting strategies, either by antibodies or graft selection (6). The conditioning regimen may be myeloablative, such as total body irradiation, or less intense, such as high-dose cyclophosphamide (1, 7). Reduced-intensity regimens are safer and usually preferred by most transplant centers to treat autoimmune diseases. However, some authors debate that myeloablative regimens may promote better control of autoreactivity and therefore longer lasting disease control (8). Finally, the previously collected cells are reinfused intravenously to the patient and warrant reconstitution of the immune system. These cells may be administered unselected or undergo CD34^+^ selection, which is also debated. While unselected grafts may increase the risk of reinfusing autoreactive cells and perpetuating the autoimmune disorder, selected grafts delay immune reconstitution and may increase the risk of viral infections (9, 10). Following autologous cell infusion, neutrophil engraftment usually occurs within two weeks, which is very similar to the time for engraftment of neutrophils following autologous and allogeneic transplants for conventional hematological indications using peripheral blood as source for hematopoietic stem and progenitor cells (11–13). In most cases, the newly recovered immune system is self-tolerant and allows control of the disease activity without further immunosuppressant drugs.

Immune monitoring studies have shown profound and long-lasting changes in the immune system of patients with autoimmune diseases treated with auto-HSCT (14–20). **Table 1** summarizes the main clinical studies on multiple sclerosis, systemic lupus erythematosus, type 1 diabetes, idiopathic juvenile arthritis, and Crohn’s disease that address how the autoimmune pathology is affected by auto-HSCT. Additional mechanisms, specific to auto-HSCT in SSc patients, will be discussed later in this review. Collectively, these studies show a modulation of the inflammatory and autoreactive profile, reactivation of thymic function, increased diversity of the T cell receptor (TCR) repertoire, and improvement of regulatory mechanisms after auto-HSCT (21–38). Importantly, these studies show that patients who reactivate the autoimmune disease after auto-HSCT have a different post-transplant immune profile than those with sustained clinical remission. Multiple sclerosis patients who fail to respond to auto-HSCT present less diversity in the T cell receptor (TCR) repertoire early in the immune reconstitution process, when compared to patients that remain in remission (17). Similarly, SSc patients who reactivate the disease after auto-HSCT have lower regulatory T and B cell counts, less TCR repertoire diversity, and lower PD-1 expression on lymphocytes when compared to patients with sustained disease control (20, 39). Type 1 diabetes patients that remain insulin-free for longer periods after auto-HSCT have improvement of the immunoregulatory cell frequencies, not detected in patients with shorter insulin independency (31).

**Table 1:** Main clinical studies on autologous HSCT for autoimmune diseases (systemic sclerosis excluded) addressing mechanisms.

Diseases	References	Study design	Clinical evidence	Immune mechanism
Multiple Sclerosis	Muraro et al. (2005) (21)	7 patients 2-year follow-up	Long-lasting clinical remission	Reactivation of thymic function (RTEs) Renewal of the TCR repertoire
	Darlington et al. (2013) (22)	14 patients 2-year follow-up	Long-lasting clinical remission Abrogation of new disease activity	Reactivation of thymic function (RTEs, TRECs) Diminished capacity for Th17 responses Transient increase in FOXP3^+^ T cells
	Abrahamsson et al. (2013) (23)	12 patients 2-year follow-up	EDSS improvement	Increase in FOXP3^+^ cells and CD56^high^ natural killer cells Depletion of pro-inflammatory CD8^+^cells subsets
	Muraro et al. (2014) (24)	24 patients 1-year follow-up	Disease control (remission/relapse)	New repertoire of CD4^+^cells and clonal expansion CD8^+^cells
	de Paula Souza et al. (2015) (25)	16 patients 2-year follow-up	EDSS improvement	Normalization of gene expression in CD8^+^and CD4^+^ T cells
	Arruda et al. (2015) (26)	24 patients 2-year follow-up	EDSS improvement	Increase in FOXP3^+^ cells and expression of CTLA-4 and GITR on CD4^+^CD25^high^ T cells Modulation of immunoregulatory genes Homeostatic proliferation
	Cull et al. (2017) (27)	13 patients 2-year follow-up	EDSS stabilization 69% progression-free survival at 3 years	Reactivation of thymic function Decrease in T-regulatory cells Transient decrease in Th17 cells
Type 1 Diabetes	Li et al. (2012) (28)	13 patients 31 to 54-month follow-up	Reduced doses of insulin Reduced levels of glycosylated hemoglobin	Reduced levels of serum autoantibodies Reduced levels of IL-1, IL-17 and TNF-α Recovery of lymphocyte subsets
	de Oliveira et al. (2012) (29)	14 patients 1-year follow-up	Insulin-free remission GAD65 levels	Modulation of pro-apoptotic genes
	Zhang et al. (2012) (30)	9 patients 12-month follow-up	Insulin-free remission	Recovery of lymphocyte subsets Modulation of T cell-related genes
	Malmegrim et al. (2017) (31)	21 patients 72-month follow-up	Long-term insulin-free remission Increase in C-peptide levels	Thymic reactivation (TRECs) Increased TCR diversity Decreased effector-memory CD4+ T cells Expansion of immunoregulatory T cells Decreased frequencies of islet-specific autoreactive CTLs
	Ye et al. (2017) (32)	18 patients 12-month follow-up	Decrease in anti-GAD levels Increase in C-peptide levels Reduced doses of insulin	Reduced Th1 and Th17 cell frequencies Changes in cytokine patterns Modulation of regulatory genes
Systemic Lupus Erythematosus	Alexander et al. (2008) (33)	7 patients 96-month follow-up	Clinical remission Decreased ANA titers	Thymic reactivation Increased TCR diversity Regeneration of FoxP3^+^ T cells Recovery of CD19 B cell subsets
	Zhang et al. (2009) (34)	15 patients 8-year follow-up	Clinical remission	Sustained elevation of FoxP3^+^ T cells
Juvenile Idiopathic Arthritis	de Klee et al. (2006) (35)	12 patients 2-year follow-up	–	Restoration CD4^+^CD25^high^ T cell Reprogramming of autoreactive T cells
	Brinkman et al (2007) (36)	22 patients 80 months follow-up	Clinical remission	Recovery of lymphocyte subsets
	Wu et al. (2014) (37)	5 patients 3-year follow-up	Clinical remission	TCR diversity
Crohn’s Disease	Corraliza et al. (2015) (38)	18 patients One-year follow-up	50% endoscopic drug-free remission	Expansion of naive B cells in the blood and intestinal mucosa. Intestinal T cell depletion correlating with mucosal healing (endoscopic remission)

RTE, recent thymic emigrants; TCR, T cell receptor repertoire; TREC, T-cell receptor excision circles; EDSS, Expanded Disability Status Scale; FOXP3, forkhead box P3; GITR, Glucocorticoid-induced TNFR related protein; GAD65, glutamic acid decarboxylase; CTL, Cytotoxic T lymphocytes; ANA, antinuclear antibodies.

These evaluations are essential to understanding mechanisms and indicate possible pathways to be improved in the clinic. This review revisits the immune monitoring studies after auto-HSCT, specifically in SSc patients, and how they relate to clinical outcomes.

## Clinical outcomes of systemic sclerosis patients after transplantation

Systemic sclerosis is a chronic autoimmune disease with complex pathogenesis that includes diffuse microvasculopathy, fibrosis, and inflammation (40). Skin fibrosis is the hallmark of the disease, but internal organ involvement, mainly heart and lungs, is frequent and usually associated with a poor prognosis (41). Conventional treatment is based mainly on immunosuppressive and vasodilator approaches, with antifibrotics paving the way more recently, and has modest benefit in controlling disease progression (42). Patients with severe and progressive disease benefit from auto-HSCT, and SSc is a growing indication in the field. According to international registries, the number of SSc patients that undergo auto-HSCT has nearly doubled in the past ten years (1), and patient outcomes have improved (43).

Over the years, knowledge about patient selection and intra-transplant management has significantly reduced transplant-related mortality to the current rate of 3 to 5%. Nevertheless, SSc is still an autoimmune disease with high transplant-related mortality, primarily due to baseline organ involvement, mainly the heart (44). Recent strategies to decrease cardiac toxicity associated with auto-HSCT include extensive cardiac evaluations before enrolling for transplant and conditioning regimens with lower doses of cyclophosphamide, a drug known to potentially damage the heart (43, 45). Further studies will show if such interventions impact long-term patient outcomes.

Since the early 2000s, phase I and II studies have shown the potential of auto-HSCT to reverse skin involvement and at least stabilize interstitial lung disease in patients with SSc (46–52). There is a significant reduction of the modified Rodnan’s skin score (mRSS), used to clinically quantify the extent and severity of cutaneous involvement, mainly in the first year after auto-HSCT. The pulmonary function also ceases to decline after the procedure, indicating stabilization of lung disease, and some studies were even able to show improvement of forced vital capacity measurements (52–54). Moreover, patients increase their quality of life and functional capacity measured by the six-minute walk test after auto-HSCT, independence and well-being indicators (55, 56). Three randomized-controlled trials plus a non-randomized comparative study have proven the superiority of auto-HSCT over standard treatment with intravenous cyclophosphamide pulses in improving overall survival, progression-free survival, and quality of life (53, 57–59). These critical studies show that, unlike conventional treatment, auto-HSCT can change the course of the disease.

However, although patient outcomes have improved after auto-HSCT, a few questions remain unanswered. SSc-reactivation over the 5 to 7 years that follow auto-HSCT is estimated at approximately 20% of patients (44, 52, 57, 58, 60). We cannot predict or early detect patients that will reactivate the disease after auto-HSCT. Additional immune reconstitution studies are essential to answer these and other questions (**Table 2**).

**Table 2:** Overview of studies that evaluated immune reconstitution and clinical outcomes in SSc patients treated with auto-HSCT.

References	Number of patients	Duration of follow-up (mo)	Biological samples	Clinical evaluation	Laboratory analyses	Clinical association
Storek et al. (2004) (61)	30	1, 3, 6, 12, and 24	PBMC	Infection rates	Antibody levels CDR3 spectratyping Immunophenotyping Thymic size	Infection rates
Farge et al. (2005) (14)	7	3, 6, 9 and 12	PBMC	Cardiac and renal function HAQ mRSS	CDR3 spectratyping Immunophenotyping TRECs assay	Response or a relapse of disease
Bohgaki et al. (2009) (62)	10	3, 6 and 12	PBMC Serum	Cardiac, pulmonary, renal function mRSS	Antibody levels Foxp3 mRNA levels Immunophenotyping sjTREC assay	Response or a relapse of disease
Fleming et al. (2008) (63)	7	Until 72	Skin biopsies	Capillary counts, mRSS, MHAQ	Immunohistochemistry and mRNA *in situ* hybridization	–
Tsukamoto et al(2011) (64)	11	1, 3, 6, 12, 24 and 36	PBMC Serum	mRSS DLCO Kl-6 SP-D	Antibody levels Cytokine levels Immunophenotyping	mRSS
Baraut et al. (2014) (18)	7	24	PBMC	mRSS	Immunophenotyping suppressive capacity assay	–
Michel et al (2016) (65)	20	6, 12, 24, 36 and 48	Serum	mRSS	Cytokine levels	–
Farge et al (2017) (66)	10	24, 36, 48, 60, and 72	PBMC Serum	FVC mRSS	Antibody levels Immunophenotyping TCR repertoire	Response or a relapse of disease
Arruda et al. (2018) (39)	31	6, 12, 24 and 36	PBMC Serum	mRSS C-reactive protein	Antibody levels Cytokine levels Immunophenotyping Quantification of sjTREC, βTREC and Cj and sjKREC TCR repertoire	Response or a relapse of disease
Arruda et al (2018) (67)	25	6, 12, 24 and 36	PBMC Serum	mRSS Lung, Gastrointestinal and renal involvement C-reactive protein	Antibody levels Cytokines levels Immunophenotyping Quantification of telomere length	Response or a relapse of disease
Assassi et al. (2019) (68)	62	8 and 26	Whole blood Serum	FVC mRSS	Gene expression profiling Serum protein composite score	FVC mRSS
Gernert et al (2019) (69)	6	1, 2, 3, 5-7, 12-16	PBMC	–	Immunophenotyping Cytokines measuring	–
Gernet et al (2020) (70)	17	4-14	Whole blood	mRSS Lungs and heart fuction	Immunophenotyping	Infectious complications
Arruda et al. (2020) (71)	8	18 (mean)	PBMC	mRSS Lung function	TCR diversity Frequency of CMV-specific clonotypes	Responder/non-responders/relapse
Lima-Júnior et al. (2021) (20)	22	1, 2, 3, 6, and 12	PBMC Serum	mRSS Lungs, heart, kidney and gastrointestinal tract function	Antibody levels Cytokines levels Immunophenotyping Suppressive capacity assay Signaling pathways	Responder/non-responders/relapse
Santana-Gonçalves et al. (2022) (72)	27	0,6,12,18,24,30 and 36	Serum Skin biopsies	mRSS Lungs, heart, kidney, gastrointestinal tract function and vascular involvement.	Cytokines levels Immunostaining in skin biopsies	Severity disease
Zanin-Silva et al. (2022) (73)	39	0 and 12	Serum Skin biopsies	mRSS Lungs, heart, kidney and gastrointestinal tract function	Cytokines levels Immunostaining in skin biopsies	Severity disease

PBMC, peripheral blood mononuclear cells; HAQ, Health Assessment Questionnaire; SP-D, surface protein D (SP-D); MHAQ, Modified Health Assessment Questionnaire Disability Index; mo, months; mRSS, modified Rodnan Skin Score.

## Immunological outcomes of systemic sclerosis patients after auto-HSCT

### Reconstitution of innate immune cells in SSc after auto-HSCT

The innate immune system has a critical role in SSc pathogenesis (74, 75). Neutrophils from SSc patients exhibit distinct phenotypic and functional changes, such as deficiencies in cell migration, phagocytosis, and chemokine receptor expression (76). Monocytes are also in disbalance in dcSSc (diffuse cutaneous systemic sclerosis) since high numbers of circulating CD16^+^ monocytes have been detected and correlated with the severity of skin fibrosis (77). These cells further display the potential to differentiate into myofibroblasts, the primary cell type responsible for depositing extracellular matrix components (ECM) and fibrosis (78). Likewise, there are increased numbers of circulating natural killer (NK) cells with activated phenotype in dcSSc, and they produce high levels of IL-6 under stimulation (79). Differently, plasmacytoid dendritic cells (pDC) are reduced in the blood of SSc patients but accumulate in the skin and lungs, correlating with inflammation, leukocyte migration, and wound repair protein levels (80).

**Figure 1:**
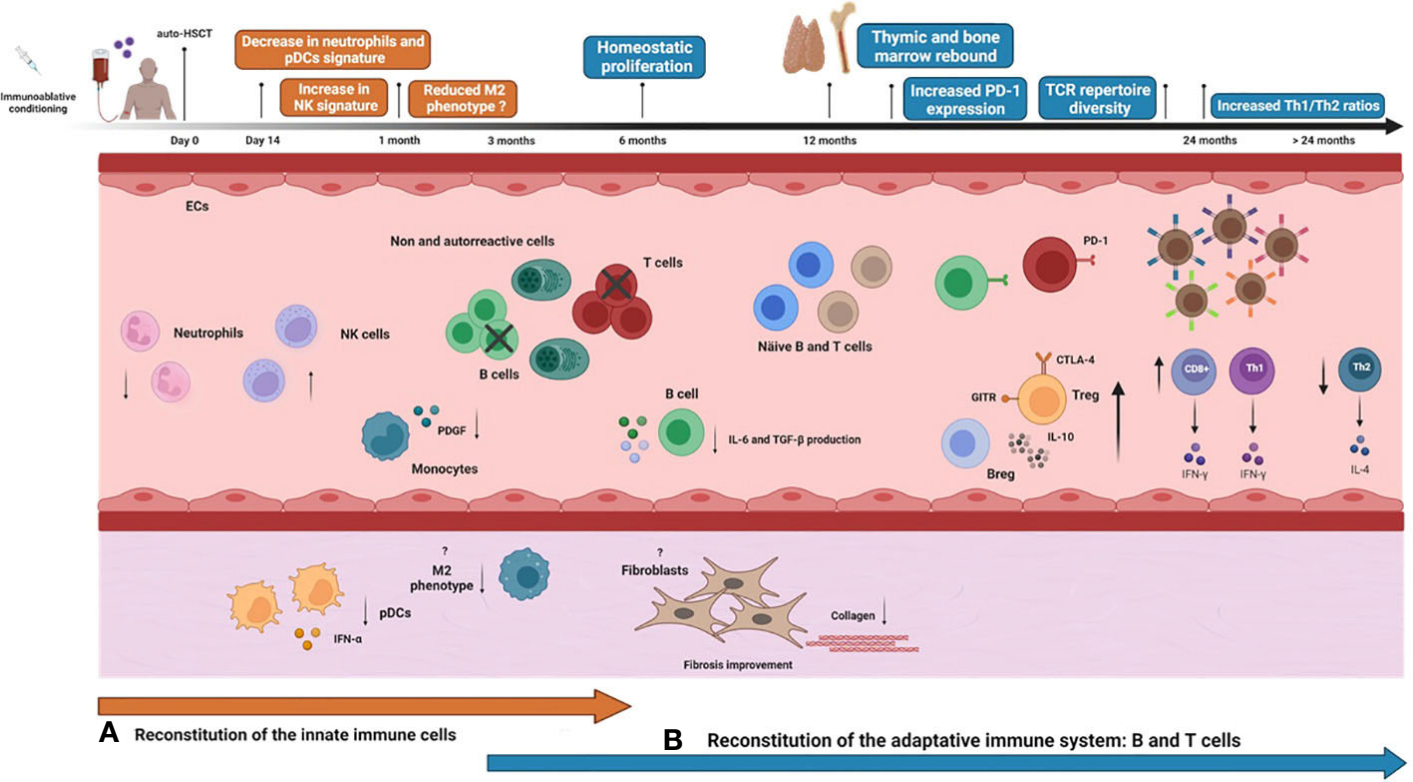
Immune reconstitution over time after auto-HSCT in systemic sclerosis patients. Systemic sclerosis (SSc) patients undergoing autologous hematopoietic stem cell transplantation (auto-HSCT) are treated with an immunoablative conditioning regimen consisting of high doses of chemotherapy/radiotherapy/immunotherapy agents, usually cyclophosphamide plus anti-thymocyte globulin. Then, previously collected autologous hematopoietic stem and progenitor cells (HSPCs) are thawed and administered to the patient intravenously. The graft may be CD34^+^ selected or non-manipulated, according to institutional protocols. After a period of bone marrow aplasia, there is hematological and immunological recovery, and the innate immune system recovers earlier than the adaptive system. Neutrophils are the first immune cell type to reconstitute, generally within the first 14 days after infusion of the HSPC graft. Neutrophil gene expression signatures significantly decrease after auto-HSCT. Other innate immune cells, such as monocytes, natural killer (NK) cells, and dendritic cells (DCs), achieve normal counts within the first month after transplantation. At this point, increased transcription signature of circulating NK cells is reported. In parallel, SSc skin biopsies post-auto-HSCT show significant reductions in mRNA expressions of plasmacytoid dendritic cells (pDC) and IFN-α responses. Alternatively-activated monocytes with an M2 phenotype significantly diminish post-transplantation. Plasma levels of platelet-derived growth factor (PDGF), an important molecule involved in fibrosis that positively correlates with the number of circulating monocytes in SSc, reduce significantly at six months after auto-HSCT. Improvement of skin fibrosis is evidenced within 6 months post-auto-HSCT, detected by decreasing modified Rodnan Score (mRSS) scores and reduced collagen deposition in the skin of SSc patients. Whether auto-HSCT affects the phenotype and function of fibroblasts from SSc patients is still unknown. B and T cells start to recover within the first six months after auto-HSCT. Early after transplantation, there is homeostatic proliferation, a process in which both cell subtypes expand in response to transplant-induced lymphopenia from residual cells that were not entirely depleted by the conditioning regimen or from cells that were re-infused with the graft. During the first-year post-transplantation, PD-1 expression is transiently increased on T and B cells as an important mechanism to control homeostatic activation. Circulating profibrotic IL-6 and TGF-β1-producing B cell subsets transiently decrease at 6 and 12 months after auto-HSCT, possibly contributing to the amelioration of skin fibrosis. Thymic reactivation or rebound, usually detectable beyond the first-year post-transplantation, promotes the exportation of newly generated naive T-cells, including regulatory T cells, thereby increasing the peripheral TCR repertoire diversity. In parallel to the thymic rebound, there is also a parallel bone marrow rebound, marked by increased output of newly generated naive B cells. Functional and numeric recovery of Treg and Breg cells after auto-HSCT contributes to controlling autoreactivity and reestablishing self-tolerance by cell contact-dependent mechanisms, increased expression of GITR and CTLA-4, and increased production of IL-10. Finally, after transplantation, the Th1/Th2 ratio is rebalanced in SSc patients.

Most immune monitoring studies of auto-HSCT in autoimmune diseases have focused on the reconstitution of adaptive immune cells (81, 82). Nevertheless, a clear understanding of the reconstitution of all immune cells may be essential to predicting transplantation outcomes and improving patient care (83). Innate immune cells recover earlier after auto-HSCT than those from the adaptive system (61), indicating that they contribute to the regeneration of the adaptive immune system in SSc patients after auto-HSCT **(** **Figure 1** **)** (83).

Neutrophils are the first innate cell type to reconstitute, generally within the first 14 days after auto-HSCT (81, 84). Whole blood transcriptome from SSc patients show a significant decrease in neutrophil gene expression signatures after auto-HSCT compared to baseline, which also correlates with lung function improvements. However, it remains unknown if the transcriptional changes affect cell phenotype and function (68). Indeed, knowledge about the reconstitution of neutrophils and other granulocytes after auto-HSCT in SSc patients remains to be explored.

Reconstitution of NK cells also occurs early following auto-HSCT **(** **Figure 1** **)** (61, 81). CD56^+^ NK cells emerge within the first 30 days after the transplantation procedure (81). Cytotoxic NK cells transcript signature increases in dcSSc patients after auto-HSCT and correlates with a decline in skin fibrosis measured by mRSS (68). These results suggest that the transcriptomic signatures of the innate immune cells are normalized following transplantation and indicate a possible immunomodulatory role for the innate immune system early after auto-HSCT (68). Nonetheless, viral infections during transplantation may influence the function of the reconstituted NK cells. A case report of a SSc patient showed that cytomegalovirus (CMV) pneumonia after auto-HSCT was partially due to a hyperreactive NK cell response (85).

Monocytes may also have an essential function in controlling the immune response in the new post-transplantation microenvironment. CD14^+^ monocytes from blood samples of transplanted patients with hematological/immunological diseases, collected within the first six months after auto-HSCT, suppress T cell proliferation *in vitro* compared to healthy subjects (86). In SSc patients, serum levels of chemokines and cytokines related to the alternatively activated M2 phenotype, such as CCL-18, IL-6, and MCP-1, were significantly diminished after auto-HSCT. At the same time, no changes occurred in patients treated with monthly intravenous cyclophosphamide infusions (68). In another cohort, SSc patients treated with auto-HSCT had reduced serum concentrations of platelet-derived growth factor (PDGF) (65), an important molecule involved in fibrosis, playing a central role in the expansion of ECM-producing cells (87).

Recently, van der Kroef et al. (88) showed that the number of circulating monocytes positively correlated with plasma concentrations of PDGF-BB in SSc patients. These results suggest that reductions in PDGF and other circulating pro-fibrotic mediators after auto-HSCT could be related to functional changes in monocytes and macrophages. These results highlight a possible role of monocytes in regulating T cell responses after auto-HSCT in SSc patients **(** **Figure 1** **)**. We encourage future immune monitoring studies to determine the role of monocyte subsets in SSc pathogenesis and immune regulation after auto-HSCT (83).

Considering the innate immune cells as the primary source of proinflammatory cytokines, investigations about how they contribute to T cell polarization and function in the context of auto-HSCT are essential. Auto-HSCT corrects the Th1/Th2 imbalance in SSc patients (64), but it is still unknown if the innate system contributes to this shift and which cell subtypes are involved (83).

Along with immune monitoring from blood samples, analyses of tissues affected by the disease, such as skin and lungs, may provide more insights into the local effects of auto-HSCT (83). Transplantation was able to reduce collagen deposition in the skin of SSc patients (73, 89), improved the microvascular morphology (63, 72, 90) and decreased lung infiltrates (91). In addition, the expression of IFN-α and CD123^+^ (pDC markers) is associated with fibrosis in dcSSc (92). Skin biopsies of dcSSc patients showed significant reductions in IFN-α and CD123^+^ mRNA expression after auto-HSCT which inversely correlated with capillary numbers in the skin (63). These results demonstrate the positive effects of auto-HSCT on pDC phenotype and IFN-α response, with implications on microvasculature and fibrosis outcomes **(** **Figure 1** **)** (83).

A remaining question is how the auto-HSCT rebalances the communication/interaction between innate immune cells, endothelial cells, and fibroblasts, which could explain the positive effects of transplantation in the fibrosis and vasculopathy pathological axes (83). SSc patients treated with auto-HSCT have improved microvascular morphology and significantly reduced endothelial activation in the dermis (72). Auto-HSCT can also induce connective tissue remodeling and decrease inflammation markers in the skin, such as S100 calcium-binding protein A9 (S100A9) and NF-κB (73). How these mechanisms interact is still to be determined.

Finally, other innate immune components involved in SSc pathogenesis, such as pattern recognition receptors (PRRs) signaling pathways, damage-associated molecular patterns (DAMPs), and innate lymphoid cells (75), should be investigated to provide scientific bases to understand the effects of auto-HSCT in SSc and potential biomarkers of response. Whether the recently emerged innate immune system initiates a permissive environment for the new and tolerant adaptive immune system after auto-HSCT regeneration remains elusive (83).

### Reconstitution of T cells in SSc after auto-HSCT

T cells play a critical role in the pathogenesis of SSc (93–97). These cells infiltrate the skin before any evidence of cutaneous fibrosis, suggesting their participation in the initial events of the disease (93). T cell receptors from skin infiltrates show oligoclonal repertoires, evidencing failure of tolerance mechanisms (98). Overlapping TCRβ repertoires in CD4^+^ and CD8^+^ T cells from peripheral blood of SSc patients are highly stable over time, indicating temporal persistence of a low diversity T cell repertoire (99). In this context, auto-HSCT ablates autoreactive clones of T and B cells, renews the immune system, and reestablishes immune tolerance (**Table 3**) (5, 6, 100).

**Table 3:** Immunophenotyping of peripheral blood T and B cell subsets in SSc patients undergoing auto-HSCT.

CELL SUBSET	PHENOTYPE	REFERENCES
Total CD3, CD4, CD8	CD3^+^; CD3^+^CD4; CD3^+^CD8^+^	(14, 39, 62, 64, 66)
Recent thymic emigrants	CD3^+^CD4^+^CD45RA^+^CD31^+^	(39)
Naive T cells Memory T cells	CD4^+^CD45RA^+^ CD4^+^(CD8)CD27^+^CD45RO^-^ CD4^+^CD45RO^+^	(14, 62, 64, 66) (39) (14, 64)
Central-memory T cells Effector-memory T cells Effector T cells Senescent T cells	CD4^+^(CD8)CD27^+^CD45RO^+^ CD4^+^(CD8)CD27^-^CD45RO^+^ CD4^+^(CD8)CD27^-^CD45RO^-^ CD8^+^CD28^-^CD57^+^ CD8^+^CD28^-^FoxP3^+^ CD3^+^CD4^+^(CD8)PD1^+^	(39) (39) (67) (67) (67)
Th1 Th2 Tc1 Tc2 Regulatory T cells Total CD19 cells Transitional B cells Post-switched memory B cells Pre-switched memory B cells Double-negative memory B cells Naive B cells Transitional Bregs Memory Bregs	CD3^+^CD8^-^ INFγ^+^ CD4^+^INFγ^+^ CD3^+^CD8^-^ IL-4^+^ CD4^+^IL-4^+^ CD3^+^CD8^+^ INFγ^+^ CD3^+^CD8^+^ IL-4^+^ CD4^+^CD25^+^ CD4^+^Foxp3^+^ CD4^+^CD25^hi^Foxp3^+^ CD4^+^CD25^+^TGF-β^+^ CD4^+^CD25^+^IL-10^+^ CD4^+^CD25^hi^CTLA-4^+^ CD4^+^CD25^hi^GITR^+^ CD19^+^ CD38^+^CD10^+^IgD^+^ CD19^+^CD27^+^IgD^-^ CD19^+^CD27^+^IgD^+^ CD19^+^ CD27^-^IgD^-^ CD19^+^CD27^-^IgD^+^ CD19^+^CD24^hi^CD38^hi^ CD19^+^CD24^hi^CD27^+^	(62) (64) (62) (64) (62) (62) (62, 64) (62) (18, 39) (18) (18) (39) (39) (14, 20, 39, 66, 69, 70) (69, 70) (20, 69, 70) (20, 69, 70) (20, 69, 70) (20, 39, 69, 70) (20) (20)

For the past two decades, immune monitoring studies have investigated the role of T cells in auto-HSCT for SSc and how they influence patient clinical outcomes (14, 18, 39, 61, 62, 64, 66).

### Homeostatic proliferation

After the immunosuppressive regimen of auto-HSCT, T lymphocytes can recover from two sources: expansion of residual T cells, known as homeostatic proliferation (HP), or thymopoiesis, generating new naive T cells (101). In the early post-transplantation periods, naive and memory T cells that survive the conditioning regimen or are infused with the graft expand in response to lymphopenia (101).

In SSc, CD8^+^ T cells recover earlier than CD4^+^ T cells, achieving normal levels at 1 to 3 months after auto-HSCT, regardless of the clinical outcomes after the procedure (14, 61, 62, 64). The rapid reconstitution of CD8+ T cells also occurs in other clinical scenarios of lymphopenia, such as sepsis, post-chemotherapy, and auto-HSCT for other autoimmune diseases **(** **Figure 1** **)** (102–21).

Studies report divergent results about the reconstitution of naive CD4^+^ T cells. Farge and collaborators described that the absolute number of naive T cells remained lower than baseline levels during the first nine months after auto-HSCT and reconstituted faster in patients who reactivated the disease after transplantation than those who remained in remission. Memory CD4^+^ T cell reconstitution did not differ between groups (14). Another study observed a similar trend, with naive CD4+ T cell remaining low until six months post-transplant in patients who sustained disease remission while reconstituting earlier in patients who reactivated the disease after the procedure (62). Tsukamoto et al. demonstrated that in SSc patients transplanted with CD34+ selected grafts, naive CD4^+^ T cells remained lower than baseline until 36 months, while memory CD4^+^ T cells returned to baseline levels at 24 months after auto-HSCT (64). Homeostatic proliferation after auto-HSCT was also associated with transient telomere attrition and increased senescent CD8^+^CD28^−^CD57^+^ T cells (39). These cells have immunomodulatory properties and might have a role in controlling autoimmunity early post-transplant (103). These studies show that the delayed recovery of naive CD4+ T cells is associated with favorable clinical response in SSc treated with auto-HSCT (**Figure 1** **)**.

### Increased PD-1 expression

The lymphopenia that follows the intense immunosuppression regimen of auto-HSCT leads to homeostatic proliferation and may exacerbate the expansion of residual autoreactive T cells, perpetuating the autoimmune disease (104). The expression of the co-inhibitory programmed death-1 (PD-1) receptor is an essential mechanism for controlling homeostatic activation in the first months after auto-HSCT and keeping autoreactive T cell clones under control (105). In SSc patients, PD-1 expression transiently increases on CD4^+^ and CD8^+^ T cells during the first-year post-transplantation. In addition, CD4^+^ and CD8^+^ T cells from patients with better clinical outcomes have higher PD1 expression than patients who reactivate the disease after auto-HSCT, indicating that this is an important immune regulatory mechanism in the early stages after transplantation (67). **(** **Figure 1** **)**. Once thymic rebound establishes itself, newly-generated naive and regulatory T cells are responsible for maintaining long-lasting self-tolerance (106).

### Thymic reactivation

Thymic involution is a physiological process that includes atrophy, loss of cells, and structural changes of the organ, mainly associated with age (107). In immune monitoring studies after transplant, TCR rearrangement excision circles (TRECs) are used to assess thymic function. TRECs are small circles of DNA formed during the rearrangement of T cell receptors that do not duplicate during mitosis (108). TREC counts in the peripheral blood reflect new T cells exported by the thymus (109, 110).

In auto-HSCT, thymic rebound is defined by functional reactivation and volumetric enlargement of the thymus after the immunosuppression regimen and re-infusion of autologous hematopoietic stem and progenitor cells (111). The thymic reactivation generally occurs after one to two years post-transplantation, depending on the patient’s age, and has been associated with favorable clinical response of autoimmune disease patients (21, 31, 31, 39). Storek et al. (61) showed an increase in thymic size detected by computed tomography at three and 12 months and increased TREC frequencies between one- and two-years post-transplantation in patients with multiple sclerosis and systemic sclerosis **(** **Figure 1** **)**.

Farge et al. showed that in patients that reactivated SSc after auto-HSCT, TREC values transiently increased from 6 to 8 months after transplantation, decreasing thereafter (14). These results corroborate those from Bohgaki et al. demonstrating significantly reduced sjTREC levels at three months after auto-HSCT in patients with good outcomes compared with those that reactivated the disease (62). Arruda et al. reported a positive correlation between sjTREC levels and frequencies of early naive T cells, named recent thymic emigrants (RTE) with the phenotype CD3^+^CD4^+^CD45RA^+^CD31^+^ in the peripheral blood of SSc patients, an alternative and complementary method to quantify thymic function. The RTE number also correlated with regulatory T cell (Treg) counts and better clinical outcomes after auto-HSCT (39).

In summary, the thymic rebound is one of the main immunological mechanisms of auto-HSCT. Efficient production of new naive T cell cells generates a diverse TCR repertoire that has been associated with the control of the autoimmune disease after auto-HSCT **(** **Figure 1** **).**

### TCR repertoire diversity

The generation of naive T cells after auto-HSCT -induced thymic reactivation directly affects TCR repertoire diversity (19). Farge et al. reported a disturbed T cell repertoire in SSc patients at baseline compared with age-matched controls, which did not change in the one-year follow-up after auto-HSCT (14). In a more extended follow-up study of six years, these authors showed late recovery of a polyclonal profile of the TCR repertoire similar to healthy individuals (66). However, there was no correlation between TCR diversity and clinical response to transplantation.

Arruda et al. demonstrated that SSc patients that reactivated the disease after auto-HSCT sustained a skewed TCR repertoire, indicating persistent autoreactivity one to two years after transplantation (39). In contrast, patients that remained in remission after transplantation presented a polyclonal TCR repertoire, similar to healthy individuals. In addition, patients that reactivated the disease after auto-HSCT presented increased frequencies of CMV-specific clones and a reduction of TCR diversity after the procedure. Opposingly, patients with good outcomes after transplantation showed an increase in TCR clonotypes specific to CMV, EBV, Influenza, and Dengue virus (71).

The renewal of the TCR repertoire following auto-HSCT has been proposed as a potential biomarker of therapeutic response (17). Patients with favorable clinical outcomes after auto-HSCT present low overlap of TCR clonotypes, reflecting the successful replacement of skewed and autoreactive T cell clones by a more polyclonal T cell repertoire (17).

### Increase of Th1/Th2 ratios

The Th1/Th2 disbalance contributes to the pathogenesis of several organ-specific and systemic autoimmune diseases (112). The Th2 response, characterized by the production of profibrotic cytokines such as IL-4, IL-6, IL-5, and IL-13, has a critical relevance in the pathogenesis of SSc (113–115). These cytokines stimulate collagen synthesis by fibroblasts and are chronically elevated in the serum of SSc patients. In contrast, the anti-fibrotic Th1 cytokine IFN-γ is reduced in SSc patients (116–119).

Frequencies of IFN-γ-producing CD8^+^ T cells increase at 12 months after transplantation, regardless of the clinical outcomes after auto-HSCT (62). Tsukamoto et al. showed that the ratio of IFN-γ/CD4^+^ to IL-4/CD4^+^ increased one month after auto-HSCT, reaching a plateau at six months that was maintained for at least 36 months. However, despite the Th1/Th2 ratio increase, there were no correlations between changes in the Th profile and modified Rodnan skin score (mRSS) (64). There are few studies evaluating the reconstitution of T cell subsets after auto-HSCT. Therefore, more studies are warranted to understand how T cell subsets contribute to the different clinical outcomes in SSc patients after auto-HSCT.

### Regulatory T cell recovery

Regulatory T cells (Tregs) expressing the transcription factor forkhead box P3 (FOXP3) represent 5–10% of the peripheral CD4^+^ T cells in humans and are crucial for the maintenance of self-tolerance and immune homeostasis (120, 121). SSc patients present decreased frequencies and reduced suppression capacity of circulating Tregs compared to healthy individuals (122–124).

Bohgaki et al. showed that CD4^+^CD25^+^ T cells increased at 12 months in SSc patients treated with unselected CD34^+^ grafts, regardless of clinical outcomes (62). FOXP3 gene expression levels did not correlate with the therapeutic response or graft selection. Another immune monitoring study of autologous transplants for SSc using selected grafts showed a severely delayed reconstitution of Tregs (64). The frequencies of CD4^+^CD25^+^ and CD4^+^FOXP3^+^ T cells remained lower than baseline even at 36 months post-transplantation **(** **Figure 1** **)** (64).

Baraut et al. evaluated the suppressive capacity of Tregs by co-culture with autologous effector T cells (18). The suppressive function of CD4^+^CD25^high^CD127^low^ Tregs was restored 24 months after auto-HSCT, along with increased numbers of IL-10-producing CD4^+^CD25^+^ Tregs. On the other hand, CD4^+^CD25^+^TGF-β T cell counts remained significantly decreased before and after auto-HSCT (18). These findings indicate an improvement of the suppressive capacity of Tregs by cell contact-dependent mechanisms and the production of the inhibitory cytokine IL-10.

Patients with good clinical response to auto-HSCT present higher CD4^+^CD25^high^FOXP3^+^ Treg percentages after the procedure than those that reactivate the disease (39). Tregs from responsive patients also show increased expression of GITR and CTLA-4 molecules compared to the patients that reactivate the disease after auto-HSCT (39).

Although the results diverge across different studies, the reported findings indicate that auto-HSCT induces a functional recovery of Treg with increased IL-10-production, improved cell contact-dependent suppression, and higher expression of GITR and CTLA-4. Effective functional Treg reconstitution has been related to favorable clinical outcomes of SSc and is currently considered another pivotal mechanism of auto-HSCT.

### Changes in serum cytokines

Several T cell-related cytokines are implicated in SSc pathogenesis and associated with fibrosis and vascular damage (18, 114, 125, 126). Auto-HSCT changes serum levels of inflammatory and pro-fibrotic cytokines by regenerating the immune system (39, 64, 65, 72, 73). High serum levels of tumor necrosis factor-alpha (TNF-α), IL-6, and soluble interleukin 2 receptor (sIL-2R) are found at baseline in the SSc patients, with a significant decrease after auto-HSCT (64). Serum levels of IL-2, IL-8, and TGF-β also transiently decrease after auto-HSCT. Still, changes in levels of the profibrotic cytokine TGF-β are not associated with the improvement of the skin score (mRSS) (55). Other cytokines, such as IL-6, IL-10, and IFN-γ, are increased at baseline and do not decrease for at least 48 months after transplantation (65).

Expression of tumor necrosis factor receptor (TNFR) was found higher on dermal T lymphocytes from SSc patients than in healthy controls. Activated peripheral blood lymphocytes also secreted more IL-6, soluble IL-6 receptor and IL-13, and induced higher type 1 collagen expression in fibroblasts compared to lymphocytes from healthy controls. In one subject that had been treated with auto-HSCT, expression of TNFR and IL-6 decreased in the dermis at the six-month time point after the procedure. These results indicate a therapeutic potential of auto-HSCT in improving the inflammation-fibrosis axis in SSc (127).

Recently, Zanin-Silva et al. observed alterations in connective tissue and fibrosis-related molecules after auto-HSCT (63). One-year post-transplant, SSc patients exhibited significant decreases in serum concentrations of platelet-derived growth factor (PDGF)-AA, PDGF-BB, tissue inhibitor of metalloproteinases (TIMP)-1 and S100A9. On the other hand, serum levels of collagen I alpha 1 (COL1A1) increased after transplantation, indicating collagen degradation (73).

Assassi et al. investigated molecular changes in the peripheral blood cell transcriptome in 62 SSc patients treated with auto-HSCT. At 26-months post-transplantation, the IFN transcript score decreased significantly, indicating a long-lasting effect. In contrast, patients treated with conventional cyclophosphamide did not present significant changes in the molecular signatures (68).

Santana-Gonçalves et al. evaluated serum levels of markers of inflammation, angiogenesis, and endothelial activation before and until 36 months post-transplant (72). IL-6, von Willebrand factor (vWF), CXC Motif Chemokine Ligand 8 (CXCL8), Endothelin-1, epidermal growth factor (EGF), VEGFA, Pentraxin-3, Intercellular Adhesion Molecule 1 (ICAM-1), E-selectin, P-selectin, Thrombomodulin and IL-18 levels were significantly higher at baseline in SSc patients when compared to healthy controls, except for ICAM-1. After auto-HSCT, all biomarkers remained stable at high levels until 36 months of follow-up, indicating persistence of the vascular injury (72).

The transplant-induced changes in serum cytokine levels associated with inflammation and fibrosis indicate an at least partial systemic modulatory effect of this therapeutic approach. However, alterations in cytokine profiles are only partially responsible for the clinical outcomes of SSc patients treated with auto-HSCT.

### Reconstitution of the B cell compartment in SSc after auto-HSCT

B cells have been widely investigated in SSc pathogenesis, especially since B cell-targeting therapies have become available. Autoreactive B cells produce autoantibodies against nuclear autoantigens, such as anti-topoisomerase I (anti-Scl-70), anti-centromere, and anti-RNA polymerase III, which are associated with different disease phenotypes (42, 128). Autoantibodies directed against endothelial cells and fibroblasts are also found in SSc patients, suggesting a contribution of B cells to tissue fibrosis and vasculopathy (129, 130).

Target organs, such as the lungs and the skin, show B cell infiltrates, underscoring the importance of these cells in disease pathogenesis (131–133). In the skin, B cell infiltrates are implicated in the early stages of the disease, preceding the establishment of fibrosis (134). This is important since B cells from SSc patients can induce the production of IL-6, TGF-β, and collagen by fibroblasts (135).

Moreover, B cells with a hyperactivated phenotype have been identified in the peripheral blood of SSc patients, with overexpression of the CD19 surface signaling molecule and correlation with autoantibody production, indicating that CD19 regulation may be functionally linked with autoantibody production in SSc (136). Meanwhile, regulatory B cells (Breg) are decreased in numbers and functionality, with deficient IL-10 production (137) and inversely correlating with disease activity (138). Additionally, Breg subpopulations from SSc patients fail to suppress CD4^+^ T cells (139).

In the context of auto-HSCT, pre-existing autoreactive B cells are depleted by the immunoablative regimen (17, 21, 140). Different studies with SSc patients have assessed the kinetics of the B cell reconstitution after auto-HSCT **(** **Figure 1** **)** (1, 14, 20, 39, 66, 69, 70, 141). Memory B cell counts increase early after auto-HSCT due to homeostatic proliferation but significantly diminish after that, while numbers of naive B cells newly produced by the bone marrow increase (14, 39). Indeed, CD19^+^IgD^+^CD27^-^ naive B cell frequencies increase early after auto-HSCT compared to baseline (20). In parallel to the thymic rebound, there is also a comparable “bone marrow rebound”, with increased output of newly generated naive B cells, which is not observed in SSc patients treated with conventional therapies (39).

A remaining and intriguing question of the auto-HSCT scenario is whether complete removal of memory B and T cells is required for full therapeutic efficacy of transplant. Incomplete immunoablation or even reinfusion of autoreactive and memory cells within the autologous stem cell graft may trigger disease reactivation (100, 142). Patients with autoimmune diseases, including SSc, have more circulating double-negative memory B cells, characterized by absent expression of CD27 and IgD (143, 144). This cell subpopulation transiently increases in the first month after auto-HSCT in SSc patients, followed by a sustained decrease in later time points (20). Combined with the expansion of switched and non-switched memory B cells after transplantation, the increase of double negative memory B cells at this stage could indicate the existence of residual B-cells, either non-depleted by the transplant conditioning regimen or re-infused within the graft (20). The transiently high expression of the negative co-stimulatory molecule PD-1 in B cells of SSc patients in these early periods after transplantation may have a role in preventing the exaggerated proliferation of autoreactive B cells (20). A similar mechanism of control is described in multiple sclerosis (MS) patients, correlating with good neurological outcomes after auto-HSCT (19).

Circulating CD19 and CD20 B cell counts are inversely associated with clinical response, suggesting that pathogenic B cell clones may preferentially expand in patients with less favorable outcomes (14, 66). Additionally, in patients with a good response to auto-HSCT, there is a sustained and positive slope of B cell reconstitution, which may reflect increased numbers of B cell subsets that promote disease control, especially those with a regulatory phenotype and function (66).

Timely reconstitution of regulatory T and B cells after transplantation is considered a key element in modulating the activation and proliferation of potent autoreactive cells after auto-HSCT (19, 39, 145). Multiple studies have shown that the T cell-mediated regulatory network improves in SSc and other autoimmune diseases following auto-HSCT (17, 21, 146). However, recent studies have also shown an increased frequency of B cells with regulatory and anti-inflammatory phenotypes in SSc patients after auto-HSCT (20, 139). IL-10 release by peripheral B cells of SSc patients post-transplantation significantly raises compared to baseline (141).

Additionally, transitional Bregs (tBregs, CD19^+^CD24^high^CD38^high^) and memory Bregs (mBregs, CD19^+^CD24^high^CD27^+^) are IL-10-producing cells (147–149) that increase after auto-HSCT (20). Patients with disease remission after auto-HSCT present higher frequencies of tBregs than those who reactivate SSc, both at baseline and 360 days post-transplant. Moreover, tBregs also recover their ability to suppress the production of Th1 cytokines by CD4^+^ T cells after auto-HSCT (20).

Previous studies have shown that phosphorylation of ERK1/2 and p38 MAPK contributes to IL-10 production (150, 151) and that SSc Bregs have impaired p38 MAPK phosphorylation pathways (135). Lima-Júnior and collaborators demonstrated that Bregs increased the phosphorylation of ERK1/2 and p38 MAPK proteins after auto-HSCT (20). Thus, an increase in ERK1/2 and p38 MAPK phosphorylation may be one of the mechanisms responsible for improving Breg suppressive function in SSc treated with auto-HSCT (20). Circulating profibrotic subsets of IL-6 and TGF-β1-producing B cells also decreased after auto-HSCT, possibly contributing to the observed amelioration of skin fibrosis (20). Future studies should investigate a possible relationship between the decreased numbers of these B cell subsets and tissue fibrosis.

We suggest that the profound alterations in the B cell compartment in SSc patients induced by auto-HSCT cannot be achieved with conventional B cell–depleting therapies. Rituximab, a monoclonal antibody against CD20 molecule, has been used to selectively deplete B-cells in many autoimmune diseases, with transient and variable responses (152–154). Treatment of rheumatoid arthritis (RA) with rituximab promotes B-cell depletion and is associated with clinical response (155). However, half of the rheumatoid arthritis patients eventually lose responsiveness over time, requiring additional rituximab infusions (154). Three-quarters of these patients recover disease control after consecutive treatment cycles with rituximab, indicating that loss of response is reversible and that patients may still improve (154). However, for type 1 diabetes (T1D) patients, rituximab was ineffective in resetting defective B cell tolerance checkpoints (153).

SSc patients treated with rituximab showed benefits in the skin (155–157) and lung involvements (158). In parallel, treatment with belimumab, another monoclonal antibody that leads to B cell apoptosis and decreases autoantibody production (159) resulted in significant improvement of mRSS in SSc patients with early disease and alterations in the expression of the profibrotic gene pathways in B cells. These studies indicate that although the B cell–depleting therapies are effective in the control activity of specific autoimmune diseases, they do not promote profound or long-lasting modifications in the patient’s immune system. Therefore, auto-HSCT may be a better therapeutic alternative than conventional B cell-directed therapies for promoting long-lasting improvements in the B-cell compartment.

## Conclusions and perspectives

In conclusion, auto-HSCT involves multiple immune mechanisms that collectively improve SSc patient clinical outcomes. Reconstituted neutrophils, monocytes, natural killers, and dendritic cells may exert an important role in producing signals that contribute to the new regulatory microenvironment promoted by auto-HSCT. However, the literature lacks studies regarding these cells and how the innate and adaptative immune systems interact after auto-HSCT. Future investigations of the innate immune cell subsets, including their phenotype, numbers, and functionality, may increase the understanding of SSc pathogenesis, perhaps even beyond the transplantation scenario.

In the context of auto-HSCT for autoimmune diseases, including SSc, the roles of homeostatic proliferation, thymic and bone-marrow rebound, T cell repertoire diversity, and increase of T and B cell-mediated regulation are currently well-described mechanisms. Nonetheless, more vigorous efforts are needed to better correlate the immunological mechanisms of auto-HSCT with patient clinical outcomes. Further cellular function assessments and comprehensive molecular analyses may identify immune signatures associated with disease remission or reactivation after auto-HSCT. Collaborative approaches to evaluate the immune profile of more significant numbers of transplanted SSc patients worldwide may provide helpful answers. We convene the academic community to pursue these research gaps and further improve clinical transplant protocols, allowing more SSc patients to benefit from this therapeutic approach.

## Author contributions

MK-V, MS-G, and DZ-S wrote the manuscript and performed the literature review. MS-G and DZ-S created the images. MS-G, KM, and MO critically revised the final version. All authors contributed to the article and approved the submitted version.

## Funding

This work was supported by the Coordenação de Aperfeiçoamento de Pessoal de Nível Superior (CAPES, finance code 001 and processes 88887.598001/2021-00 and 88887.597494/2021-00), by the São Paulo Research Foundation (FAPESP) (n° 2013/08135-2 and 2017/09420-3), and the Fundação de Apoio ao Ensino, Pesquisa e Assistência (FAEPA).

## Acknowledgments

In memory of Professor Julio César Voltarelli, who passed away on March 21, 2012. His greatest legacy was the establishment of the clinical protocols of hematopoietic stem cell transplantation studies for autoimmune diseases in Brazil.

## Conflict of interest

The authors declare that the research was conducted in the absence of any commercial or financial relationships that could be construed as a potential conflict of interest.

## Publisher’s note

All claims expressed in this article are solely those of the authors and do not necessarily represent those of their affiliated organizations, or those of the publisher, the editors and the reviewers. Any product that may be evaluated in this article, or claim that may be made by its manufacturer, is not guaranteed or endorsed by the publisher.

## References

[1] Alexander T Farge D Badoglio M Lindsay JO Muraro PA Snowden JA. Autoimmune diseases working party (ADWP) of the European society for blood and marrow transplant–tion (EBMT). hematopoietic stem cell therapy for autoimmune diseases . clinical experience and mechanisms. J Autoimmun (2018) 92:35–46. doi: 10.1016/j.jaut.2018.06.002 29934135

[2] Kowal-Bielecka O Fransen J Avouac J Becker M Kulak A Allanore Y. Update of EULAR recommendations for the treatment of systemic sclerosis. Ann Rheum Dis (2017) 76(8):1327–39. doi: 10.1136/annrheumdis-2016-209909 27941129

[3] Cohen JA Baldassari LE Atkins HL Bowen JD Bredeson C Carpenter PA. Autologous hematopoietic cell transplantation for treatment-refractory relapsing multiple sclerosis: Position statement from the American society for blood and marrow transplantation. Biol Blood Marrow Transplant (2019) 25(5):845–54. doi: 10.1016/j.bbmt.2019.02.014 30794930

[4] Oliveira MC Elias JB Moraes DA Simões BP Rodrigues M Ribeiro AAF. A review of hematopoietic stem cell transplantation for’autoimmune diseases: multiple sclerosis, systemic sclerosis and crohn's disease. position paper of the Brazilian society of bone marrow transplantation. Hematol Transfus Cell Ther (2021) 43(1):65–86. doi: 10.1016/j.htct.2020.03.002 32418777 PMC7910166

[5] Del Papa N Pignataro F Zaccara E Maglione W Minniti A. Autologous hematopoietic stem cell transplantation for treatment of systemic sclerosis. Front Immunol (2018) 9:2390. doi: 10.3389/fimmu.2018.02390 30386340 PMC6198074

[6] Swart JF Delemarre EM van Wijk F Boelens J-J Kuball J van Laar JM. Haematopoietic stem cell transplantation for autoimmune diseases. Nat Rev Rheumatol (2017) 13(4):244–56. doi: 10.1038/nrrheum.2017.7 28228650

[7] Lycke J Lenhoff S. Intensive immunosuppression followed by autologous hematopoietic stem cell transplantation for the treatment of multiple sclerosis. Ther Adv Neurol Disord (2020) 13:1756286420929467. doi: 10.1177/1756286420929467 32636931 PMC7315665

[8] Sullivan KM Goldmuntz EA Keyes-Elstein L McSweeney PA Pinckney A Welch B. Myeloablative autologous stem-cell transplantation for severe scleroderma. New Engl J Med (2018) 378(1):35–47. doi: 10.1056/NEJMoa1703327 29298160 PMC5846574

[9] Oliveira MC Labopin M Henes J Moore J Del Papa N Cras A. Does ex vivo CD34+ positive selection influence outcome after autologous hematopoietic stem cell transplantation in systemic sclerosis patients? Bone Marrow Transplant (2016) 51(4):501–5. doi: 10.1038/bmt.2015.299 26642332

[10] Ayano M Tsukamoto H Mitoma H Kimoto Y Akahoshi M Arinobu Y. CD34-selected versus unmanipulated autologous haematopoietic stem cell transplantation in the treatment of severe systemic sclerosis: a *post hoc* analysis of a phase I/II clinical trial conducted in Japan. Arthritis Res Ther (2019) 21(1):30. doi: 10.1186/s13075-019-1823-0 30670057 PMC6341635

[11] Seggewiss R Einsele H. Immune reconstitution after allogeneic transplantation and expanding options for immunomodulation: an update. Blood. (2010) 115(19):3861–8. doi: 10.1182/blood-2009-12-234096 20215642

[12] Danby R Rocha V. Improving engraftment and immune reconstitution in umbilical cord blood transplantation. Front Immunol (2014) 5:68. doi: 10.3389/fimmu.2014.00068 24605111 PMC3932655

[13] Ogonek J Kralj Juric M Ghimire S Varanasi PR Holler E Greinix H. Immune reconstitution after allogeneic hematopoietic stem cell transplantation. Front Immunol (2016) 7:507. doi: 10.3389/fimmu.2016.00507 27909435 PMC5112259

[14] Farge D Henegar C Carmagnat M Daneshpouy M Marjanovic Z Rabian C. Analysis of immune reconstitution after autologous bone marrow transplantation in systemic sclerosis. Arthritis Rheumatol (2005) 52(5):1555–63. doi: 10.1002/art.21036 15880600

[15] Muraro PA Douek DC. Renewing the T cell repertoire to arrest autoimmune aggression. Trends Immunol (2006) 27(2):61–7. doi: 10.1016/j.it.2005.12.003 16406806

[16] Alexander T Thiel A Rosen O Massenkeil G Sattler A Kohler S. Depletion of autoreactive immunologic memory followed by autologous hematopoietic stem cell transplantation in patients with refractory SLE induces long-term remission through *de novo* generation of a juvenile and tolerant immune system. Blood. (2009) 113(1):214–23. doi: 10.1182/blood-2008-07-168286 18824594

[17] Muraro PA Robins H Malhotra S Howell M Phippard D Desmarais C. T Cell repertoire following autologous stem cell transplantation for multiple sclerosis. J Clin Invest (2014) 124(3):1168–72. doi: 10.1172/JCI71691 PMC3934160 24531550

[18] Baraut J Grigore EI Jean-Louis F Khelifa SH Durand C Verrecchia F. Peripheral blood regulatory T cells in patients with diffuse systemic sclerosis (SSc) before and after autologous hematopoietic SCT: a pilot study. Bone Marrow Transplant (2014) 49(3):349–54. doi: 10.1038/bmt.2013.202 24362364

[19] Arruda LCM Clave E Moins-Teisserenc H Douay C Farge D Toubert A. Resetting the immune response after autologous hematopoietic stem cell transplantation for autoimmune diseases. Curr Res Trans Med (2016) 64(2):107–13. doi: 10.1016/j.retram.2016.03.004 27316394

[20] Lima-Júnior JR Arruda LCM Gonçalves MS Dias JBE Moraes DA Covas DT. Autologous haematopoietic stem cell transplantation restores the suppressive capacity of regulatory b cells in systemic sclerosis patients. Rheumatol (Oxford) (2021) 60(12):5538–48. doi: 10.1093/rheumatology/keab257 33724344

[21] Muraro PA Douek DC Packer A Chung K Guenaga FJ Cassiani-ingoni R. Thymic output generates a new and diverse TCR repertoire after autologous stem cell transplantation in multiple sclerosis patients. J Exp Med (2005) 201(5):805–16. doi: 10.1084/jem.20041679 PMC2212822 15738052

[22] Darlington PJ Touil T Doucet JS Gaucher D Zeidan J Gauchat D. Diminished Th17 (not Th1) responses underlie multiple sclerosis disease abrogation after hematopoietic stem cell transplantation. Ann Neurol (2013) 73:341–54. doi: 10.1002/ana.23784 23463494

[23] Abrahamsson SV Angelini DF Dubinsky AN Morel E Oh U Jones JL. Non-myeloablative autologous haematopoietic stem cell transplantation expands regulatory cells and depletes IL-17 producing mucosal-associated invariant T cells in multiple sclerosis. Brain (2013) 136(Pt 9):2888–903. doi: 10.1093/brain/awt182 PMC3754461 23864273

[24] Muraro PA Robins H Malhotra S Howell M Phippard D Desmarais C. Brief report T cell repertoire following autologous stem cell transplantation for multiple sclerosis. J Clin Invest (2014) 124:1168–72. doi: 10.1172/JCI71691DS1 PMC3934160 24531550

[25] de Paula Sousa A Malmegrim KC Panepucci RA Brum DS Barreira AA. Autologous hematopoietic stem cell transplantation reduces abnormalities in the expression of immune genes in multiple sclerosis. Clin Sci (2014) 120:111–20. doi: 10.1042/CS20140095 25116724

[26] Arruda LC Lorenzi JC Sousa AP Zanette DL Palma PV Panepucci RA. Autologous hematopoietic SCT normalizes miR-16,–155 and–142-3p expression in multiple sclerosis patients. Bone Marrow Transplant (2015) 50:380–9. doi: 10.1038/bmt.2014.277 25486582

[27] Cull G Hall D Fabis-Pedrini MJ Carroll WM Forster L Robins F. Lymphocyte reconstitution following autologous stem cell transplantation for progressive MS. Mult Scler J Exp Transl Clin (2017) 3:2055217317700167. doi: 10.1177/2055217317700167 28607754 PMC5415040

[28] Li L Shen S Ouyang J Hu Y Hu L Cui W. Autologous hematopoietic stem cell transplantation modulates Mmunocompetent cells and improves β-cell function in Chinese patients with new onset of type 1 diabetes. J Clin Endocrinol Metab (2012) 97:1729–36. doi: 10.1210/jc.2011-2188 22419704

[29] de Oliveira GL Malmegrim KC Ferreira AF Tognon R Kashima S Couri CEB. Up-regulation of fas and fasL pro-apoptotic genes expression in type 1 diabetes patients after autologous haematopoietic stem cell transplantation. Clin Exp Immunol (2012) 168:291–302. doi: 10.1111/j.1365-2249.2012.04583.x 22519592 PMC3390481

[30] Zhang X Ye L Hu J Tang W Liu R Yang M. Acute response of peripheral blood cell to autologous hematopoietic stem cell transplantation in type 1 diabetic patient. PLoS One (2012) 7:e31887. doi: 10.1371/journal.pone.0031887 22384093 PMC3285188

[31] Malmegrim KC de Azevedo JT Arruda LC Abreu JR Couri CE de Oliveira GL. Immunological balance is associated with clinical outcome after autologous hematopoietic stem cell transplantation in type 1 diabetes. Front Immunol (2017) 8:167. doi: 10.3389/fimmu.2017.00167 28275376 PMC5319960

[32] Ye L Li L Wan B Yang M Hong J Gu W. Immune response after autologous hematopoietic stem cell transplantation in type 1 diabetes mellitus. Stem Cell Res Ther (2017) 8:90. doi: 10.1186/s13287-017-0542-1 28420440 PMC5395765

[33] Alexander T Thiel A Rosen O Massenkeil G Sattler A Kohler S. Depletion of autoreactive immunologic memory followed by autologous hematopoietic stem cell transplantation in patients with refractory SLE induces long-term remission through *de novo* generation of a juvenile and tolerant immune system. Blood (2008) 113:214–23. doi: 10.1182/blood-2008-07-168286 18824594

[34] Zhang L Bertucci AM Ramsey-Goldman R Burt RK Datta SK. Regulatory T cell (Treg) subsets return in patients with refractory lupus following stem cell transplantation, and TGF–producing CD8+ treg cells are associated with immunological remission of lupus. J Immunol (2009) 183:6346–58. doi: 10.4049/jimmunol.0901773 PMC2784684 19841178

[35] de Kleer I Vastert B Klein M Teklenburg G Arkesteijn G Yung GP. Autologous stem cell transplantation for autoimmunity induces immunologic self-tolerance by reprogramming autoreactive T cells and restoring the CD4+CD25+ immune regulatory network. Blood. (2006) 107(4):1696–702. doi: 10.1182/blood-2005-07-2800 16263787

[36] Brinkman DM de Kleer IM ten Cate R van Rossum MA Bekkering WP Fasth A. Autologous stem cell transplantation in children with severe progressive systemic or polyarticular juvenile idiopathic arthritis: long-term follow-up of a prospective clinical trial. Arthritis Rheumatol (2007) 56(7):2410–21. doi: 10.1002/art.22656 17599770

[37] Wu Q Pesenacker AM Stansfield A King D Barge D Foster HE. Immunological characteristics and T-cell receptor clonal diversity in children with systemic juvenile idiopathic arthritis undergoing T-cell-depleted autologous stem cell transplantation. Immunology. (2014) 142(2):227–36. doi: 10.1111/imm.12245 PMC4008230 24405357

[38] Corraliza AM Ricart E López-García A Carme Masamunt M Veny M Esteller M. Differences in peripheral and tissue immune cell populations following haematopoietic stem cell transplantation in crohn's disease patients. J Crohns Colitis (2019) 13(5):634–47. doi: 10.1093/ecco-jcc/jjy203 PMC6486491 30521002

[39] Arruda LCM Malmegrim KCR Lima-Júnior JR Clave E Dias JBE Moraes DA. Immune rebound associates with a favorable clinical response to autologous HSCT in systemic sclerosis patients. Blood Adv (2018) 2(2):126–41. doi: 10.1182/bloodadvances.2017011072 PMC5787873 29365321

[40] Tsou PS Varga J O'Reilly S. Advances in epigenetics in systemic sclerosis: molecular mechanisms and therapeutic potential. Nat Rev Rheumatol (2021) 17(10):596–607. doi: 10.1038/s41584-021-00683-2 34480165

[41] Perelas A Silver RM Arrossi AV Highland KB. Systemic sclerosis-associated interstitial lung disease. Lancet Respir Med (2020) 8(3):304–20. doi: 10.1016/S2213-2600(19)30480-1 32113575

[42] Denton CP Khanna D. Systemic sclerosis. Lancet. (2017) 390(10103):1685–99. doi: 10.1016/S0140-6736(17)30933-9 28413064

[43] Farge D Burt RK Oliveira MC Mousseaux E Rovira M Marjanovic Z. Cardiopulmonary assessment of patients with systemic sclerosis for hematopoietic stem cell transplantation: recommendations from the European society for blood and marrow transplantation autoimmune diseases working party and collaborating partners. Bone Marrow Transplant (2017) 52(11):1495–503. doi: 10.1038/bmt.2017.56 PMC5671927 28530671

[44] Burt RK Oliveira MC Shah SJ Moraes DA Simoes B Gheorghiade M. Cardiac involvement and treatment-related mortality after non-myeloablative haemopoietic stem-cell transplantation with unselected autologous peripheral blood for patients with systemic sclerosis: a retrospective analysis. Lancet. (2013) 381(9872):1116–24. doi: 10.1016/S0140-6736(12)62114-X 23363664

[45] Burt RK Han X Quigley K Arnautovic I Shah SJ Lee DC. Cardiac safe hematopoietic stem cell transplantation for systemic sclerosis with poor cardiac function: a pilot safety study that decreases neutropenic interval to 5 days. Bone Marrow Transplant (2021) 56(1):50–9. doi: 10.1038/s41409-020-0978-2 32612255

[46] Binks M Passweg JR Furst D McSweeney P Sullivan K Besenthal C. Phase I/II trial of autologous stem cell transplantation in systemic sclerosis: procedure related mortality and impact on skin disease. Ann Rheum Dis (2001) 60(6):577–84. doi: 10.1136/ard.60.6.577 PMC1753658 11350846

[47] Henes JC Schmalzing M Vogel W Riemekasten G Fend F Kanz L. Optimization of autologous stem cell transplantation for systemic sclerosis – a single-center longterm experience in 26 patients with severe organ manifestations. J Rheumatol (2012) 39(2):269–75. doi: 10.3899/jrheum.110868 22247352

[48] Farge D Marolleau JP Zohar S Marjanovic Z Cabane J Mounier N. Autologous bone marrow transplantation in the treatment of refractory systemic sclerosis: early results from a French multicentre phase I–II study. Br J Haematol (2002) 119(3):726–39. doi: 10.1046/j.1365-2141.2002.03895.x 12437652

[49] Farge D Passweg J van Laar JM Marjanovic Z Besenthal C Finke J. Autologous stem cell transplantation in the treatment of systemic sclerosis: report from the EBMT/EULAR registry. Ann Rheum Dis (2004) 63(8):974–81. doi: 10.1136/ard.2003.011205 PMC1755096 15249325

[50] Nash RA McSweeney PA Crofford LJ Abidi M Chen C-S Godwin JD. High-dose immunosuppressive therapy and autologous hematopoietic cell transplantation for severe systemic sclerosis: long-term follow-up of the US multicenter pilot study. Blood. (2007) 110(4):1388–96. doi: 10.1182/blood-2007-02-072389 PMC1939909 17452515

[51] Oyama Y Barr WG Statkute L Corbridge T Gonda EA Jovanovic B. Autologous non-myeloablative hematopoietic stem cell transplantation in patients with systemic sclerosis. Bone Marrow Transplant (2007) 40(6):549–55. doi: 10.1038/sj.bmt.1705782 17646844

[52] Henrique-Neto Á Vasconcelos MYK Dias JBE de Moraes DA Gonçalves MS Zanin-Silva DC. Hematopoietic stem cell transplantation for systemic sclerosis: Brazilian experience. Adv Rheumatol (2021) 61(1):9. doi: 10.1186/s42358-021-00166-8 33549135

[53] Burt RK Shah SJ Dill K Grant T Gheorghiade M Schroeder J. Autologous non-myeloablative haemopoietic stem-cell transplantation compared with pulse cyclophosphamide once per month for systemic sclerosis (ASSIST): an open-label, randomised phase 2 trial. Lancet. (2011) 378(9790):498–506. doi: 10.1016/S0140-6736(11)60982-3 21777972

[54] Shouval R Furie N Raanani P Nagler A Gafter-Gvili A. Autologous hematopoietic stem cell transplantation for systemic sclerosis: A systematic review and meta-analysis. Biol Blood Marrow Transplant (2018) 24(5):937–44. doi: 10.1016/j.bbmt.2018.01.020 29374527

[55] Costa-Pereira KR Guimarães AL Moraes DA Dias JBE Garcia JT de Oliveira-Cardoso EA. Hematopoietic stem cell transplantation improves functional outcomes of systemic sclerosis patients. J Clin Rheumatol (2020) 26(7S Suppl 2):S131–8. doi: 10.1097/RHU.0000000000001117 31397762

[56] Puyade M Maltez N Lansiaux P Pugnet G Roblot P Colmegna I. Health-related quality of life in systemic sclerosis before and after autologous haematopoietic stem cell transplant-a systematic review. Rheumatol (Oxford) (2020) 59(4):779–89. doi: 10.1093/rheumatology/kez300 31504944

[57] van Laar JM Farge D Sont JK Naraghi K Marjanovic Z Larghero J. Autologous hematopoietic stem cell transplantation vs intravenous pulse cyclophosphamide in diffuse cutaneous systemic sclerosis: a randomized clinical trial. JAMA. (2014) 311(24):2490–8. doi: 10.1001/jama.2014.6368 25058083

[58] Sullivan KM Goldmuntz EA Keyes-Elstein L McSweeney PA Pinckney A Welch B. Myeloablative autologous stem-cell transplantation for severe scleroderma. New Engl J Med (2018) 378(1):35–47. doi: 10.1056/NEJMoa1703327 29298160 PMC5846574

[59] Del Papa N Onida F Zaccara E Saporiti G Maglione W Tagliaferri E. Autologous hematopoietic stem cell transplantation has better outcomes than conventional therapies in patients with rapidly progressive systemic sclerosis. Bone Marrow Transplant (2017) 52(1):53–8. doi: 10.1038/bmt.2016.211 27548467

[60] Ait Abdallah N Wang M Lansiaux P Puyade M Berthier S Terriou L. Long term outcomes of the French ASTIS systemic sclerosis cohort using the global rank composite score. Bone Marrow Transplant (2021) 56(9):2259–67. doi: 10.1038/s41409-021-01355-1 34108673

[61] Storek J Zhao Z Lin E Berger T McSweeney PA Nash RA. Recovery from and consequences of severe iatrogenic lymphopenia (induced to treat autoimmune diseases). Clin Immunol (2004) 113(3):285–98. doi: 10.1016/j.clim.2004.07.006 PMC2956741 15507394

[62] Bohgaki T Atsumi T Bohgaki M Furusaki A Kondo M Sato-Matsumura KC. Immunological reconstitution after autologous hematopoietic stem cell transplantation in patients with systemic sclerosis: Relationship between clinical benefits and intensity of immunosuppression. J Rheumatol (2009) 36(6):1240. doi: 10.3899/jrheum.081025 19447934

[63] Fleming JN Nash RA McLeod DO Fiorentino DF Shulman HM Connolly MK. Capillary regeneration in scleroderma: Stem cell therapy reverses phenotype? PLoS One (2008) 3(1):e1452. doi: 10.1371/journal.pone.0001452 18197262 PMC2175530

[64] Tsukamoto H Nagafuji K Horiuchi T Mitoma H Niiro H Arinobu Y. Analysis of immune reconstitution after autologous CD34+ stem/progenitor cell transplantation for systemic sclerosis: predominant reconstitution of Th1 CD4+ T cells. Rheumatology. (2011) 50(5):944–52. doi: 10.1093/rheumatology/keq414 21172925

[65] Michel L Farge D Baraut J Marjanovic Z Jean-Louis F Porcher R. Evolution of serum cytokine profile after hematopoietic stem cell transplantation in systemic sclerosis patients. Bone Marrow Transplant (2016) 51(8):1146–9. doi: 10.1038/bmt.2016.77 27042845

[66] Farge D Arruda LCM Brigant F Clave E Douay C Marjanovic Z. Long-term immune reconstitution and T cell repertoire analysis after autologous hematopoietic stem cell transplantation in systemic sclerosis patients. J Hematol Oncol (2017) 10(1):21. doi: 10.1186/s13045-016-0388-5 28103947 PMC5244700

[67] Arruda LCM Lima-Júnior JR Clave E Moraes DA Douay C Fournier I. Homeostatic proliferation leads to telomere attrition and increased PD-1 expression after autologous hematopoietic SCT for systemic sclerosis. Bone Marrow Transplant (2018) 53(10):1319–27. doi: 10.1038/s41409-018-0162-0 29670207

[68] Assassi S Wang X Chen G Goldmuntz E Keyes-Elstein L Ying J. Myeloablation followed by autologous stem cell transplantation normalises systemic sclerosis molecular signatures. Ann Rheum Dis (2019) 78(10):1371–8. doi: 10.1136/annrheumdis-2019-215770 PMC7167108 31391177

[69] Gernert M Tony H-P Schwaneck EC Gadeholt O Schmalzing M. Autologous hematopoietic stem cell transplantation in systemic sclerosis induces long-lasting changes in b cell homeostasis toward an anti-inflammatory b cell cytokine pattern. Arthritis Res Ther (2019) 21(1):106. doi: 10.1186/s13075-019-1889-8 31036055 PMC6489316

[70] Gernert M Tony HP Schwaneck EC Fröhlich M Schmalzing M. Low b cell counts as risk factor for infectious complications in systemic sclerosis after autologous hematopoietic stem cell transplantation. Arthritis Res Ther (2020) 22(1):183. doi: 10.1186/s13075-020-02255-3 32771029 PMC7414656

[71] Arruda LCM Clave E Douay C Lima-Júnior JR Slavov SN Malmegrim KCR. CMV-specific clones may lead to reduced TCR diversity and relapse in systemic sclerosis patients treated with AHSCT. Rheumatology. (2020) 59(9):e38–40. doi: 10.1093/rheumatology/keaa001 31998954

[72] Santana-Gonçalves M Zanin-Silva D Henrique-Neto Á Moraes DA Kawashima-Vasconcelos MY Lima-Júnior JR. Autologous hematopoietic stem cell transplantation modifies specific aspects of systemic sclerosis-related microvasculopathy. Ther Adv Musculoskelet Dis (2022) 14:1759720X221084845. doi: 10.1177/1759720X221084845 PMC8966069 35368373

[73] Zanin-Silva DC Santana-Gonçalves M Kawashima-Vasconcelos MY Lima-Júnior JR Dias JBE Moraes DA. Autologous hematopoietic stem cell transplantation promotes connective tissue remodeling in systemic sclerosis patients. Arthritis Res Ther (2022) 24(1):95. doi: 10.1186/s13075-022-02779-w 35488348 PMC9052524

[74] Brown M O'Reilly S. Innate immunity and toll-like receptor signaling in the pathogenesis of scleroderma: advances and opportunities for therapy. Curr Opin Rheumatol (2018) 30(6):600–5. doi: 10.1097/BOR.0000000000000542 30234721

[75] Laurent P Sisirak V Lazaro E Richez C Duffau P Blanco P. Innate immunity in systemic sclerosis fibrosis: Recent advances. Front Immunol (2018) 9:1702. doi: 10.3389/fimmu.2018.01702 30083163 PMC6064727

[76] Impellizzieri D Egholm C Valaperti A Distler O Boyman O. Patients with systemic sclerosis show phenotypic and functional defects in neutrophils. Allergy. (2021). doi: 10.1111/all.15073 PMC9293168 34467524

[77] Lescoat A Lecureur V Roussel M Sunnaram BL Ballerie A Coiffier G. CD16-positive circulating monocytes and fibrotic manifestations of systemic sclerosis. Clin Rheumatol (2017) 36(7):1649–54. doi: 10.1007/s10067-017-3597-6 28293753

[78] Binai N O’Reilly S Griffiths B van Laar JM Hügle T. Differentiation potential of CD14+ monocytes into myofibroblasts in patients with systemic sclerosis. . PLoS One (2012) 7(3):e33508. doi: 10.1371/journal.pone.0033508 22432031 PMC3303833

[79] Horikawa M Hasegawa M Komura K Hayakawa I Yanaba K Matsushita T. Abnormal natural killer cell function in systemic sclerosis: Altered cytokine production and defective killing activity. J Invest Dermatol (2005) 125(4):731–7. doi: 10.1111/j.0022-202X.2005.23767.x 16185273

[80] Kafaja S Divekar A Valera I Khanna D Saggar R Furst D. Plasmacytoid dendritic cells correlate with fibrosis in patients with systemic sclerosis and contribute to fibrosis in a murine model (P3133). J Immunol (2013) 190(1 Supplement):43.28.

[81] Szodoray P Varoczy L Papp G Barath S Nakken B Szegedi G. Immunological reconstitution after autologous stem cell transplantation in patients with refractory systemic autoimmune diseases. Scandinavian J Rheumatol (2012) 41(2):110–5. doi: 10.3109/03009742.2011.606788 21936606

[82] Malmegrim KCR Lima-Júnior JR Arruda LCM de Azevedo JTC de Oliveira GLV Oliveira MC. Autologous hematopoietic stem cell transplantation for autoimmune diseases: From mechanistic insights to biomarkers. Front Immunol (2018) 9:2602. doi: 10.3389/fimmu.2018.02602 30505303 PMC6250746

[83] Servaas NH Spierings J Pandit A van Laar JM. The role of innate immune cells in systemic sclerosis in the context of autologous hematopoietic stem cell transplantation. Clin Exp Immunol (2020) 201(1):34–9. doi: 10.1111/cei.13419 PMC7290088 31990046

[84] Stern L McGuire H Avdic S Rizzetto S Fazekas de St Groth B Luciani F. Mass cytometry for the assessment of immune reconstitution after hematopoietic stem cell transplantation. Front Immunol (2018) 9:1672. doi: 10.3389/fimmu.2018.01672 30093901 PMC6070614

[85] Chizzolini C Vukicevic M Rochat T Tyndall A Roosnek E. Acute natural killer cell pneumonia in a patient transplanted with autologous haematopoietic stem cells for systemic sclerosis. Rheumatology. (2013) 52(5):954–6. doi: 10.1093/rheumatology/kes276 23104981

[86] Hainz U. Monocyte-mediated T-cell suppression and augmented monocyte tryptophan catabolism after human hematopoietic stem-cell transplantation. Blood. (2005) 105(10):4127–34. doi: 10.1182/blood-2004-05-1726 PMC1895091 15677560

[87] Trojanowska M. Role of PDGF in fibrotic diseases and systemic sclerosis. Rheumatology. (2008) 47(Supplement 5):v2–4. doi: 10.1093/rheumatology/ken265 18784131

[88] van der Kroef M Carvalheiro T Rossato M de Wit F Cossu M Chouri E. CXCL4 triggers monocytes and macrophages to produce PDGF-BB, culminating in fibroblast activation: Implications for systemic sclerosis. J Autoimmunity (2020) 111:102444. doi: 10.1016/j.jaut.2020.102444 32284212

[89] Verrecchia F Laboureau J Verola O Roos N Porcher R Bruneval P. Skin involvement in scleroderma–where histological and clinical scores meet. Rheumatology. (2007) 46(5):833–41. doi: 10.1093/rheumatology/kel451 17255134

[90] Miniati I Guiducci S Conforti ML Rogai V Fiori G Cinelli M. Autologous stem cell transplantation improves microcirculation in systemic sclerosis. Ann Rheum Dis (2009) 68(1):94–8. doi: 10.1136/ard.2007.082495 18308744

[91] Launay D Marjanovic Z de BAZELAIRE C Florea L Zohar S Keshtmand H. Autologous hematopoietic stem cell transplant in systemic sclerosis: Quantitative high resolution computed tomography of the chest scoring. J Rheumatol (2009) 36(7):1460–3. doi: 10.3899/jrheum.081212 19531757

[92] Ah Kioon MD Tripodo C Fernandez D Kirou KA Spiera RF Crow MK. Plasmacytoid dendritic cells promote systemic sclerosis with a key role for TLR8. Sci Transl Med (2018) 10(423):eaam8458. doi: 10.1126/scitranslmed.aam8458 29321259 PMC9865429

[93] Kalogerou A. Early T cell activation in the skin from patients with systemic sclerosis. Ann Rheumatic Diseases (2005) 64(8):1233–5. doi: 10.1136/ard.2004.027094 PMC1755597 16014686

[94] Yang C Lei L Pan J Zhao C Wen J Qin F. Altered CD4+ T cell and cytokine levels in peripheral blood and skin samples from systemic sclerosis patients and IL-35 in CD4+ T cell growth. Rheumatology. (2021). doi: 10.1093/rheumatology/keab359 33878182

[95] Li T Ortiz-Fernández L Andrés-León E Ciudad L Javierre BM López-Isac E. Epigenomics and transcriptomics of systemic sclerosis CD4+ T cells reveal long-range dysregulation of key inflammatory pathways mediated by disease-associated susceptibility loci. Genome Med (2020) 12(1):81. doi: 10.1186/s13073-020-00779-6 32977850 PMC7519528

[96] Maehara T Kaneko N Perugino CA Mattoo H Kers J Allard-Chamard H. Cytotoxic CD4+ T lymphocytes may induce epithelial cell apoptosis in systemic sclerosis. J Clin Invest (2020). doi: 10.1172/JCI131700 PMC7190971 31990684

[97] Almanzar G Schmalzing M Klein M Hilligardt D Morris P Höfner K. Memory CD4+ T cells lacking expression of CCR7 promote pro-inflammatory cytokine production in patients with diffuse cutaneous systemic sclerosis. Eur J Dermatol (2019) 29(5):468–76. doi: 10.1684/ejd.2019.3645 31789272

[98] Sakkas LI Xu B Artlett CM Lu S Jimenez SA Platsoucas CD. Oligoclonal T cell expansion in the skin of patients with systemic sclerosis. J Immunol (2002) 168(7):3649–59. doi: 10.4049/jimmunol.168.7.3649 11907131

[99] Servaas NH Zaaraoui-Boutahar F Wichers CGK Ottria A Chouri E Affandi AJ. Longitudinal analysis of T-cell receptor repertoires reveals persistence of antigen-driven CD4+ and CD8+ T-cell clusters in systemic sclerosis. J Autoimmunity (2021) 117:102574. doi: 10.1016/j.jaut.2020.102574 33307312

[100] Farge D Labopin M Tyndall A Fassas A Mancardi GL Van Laar J. Autologous hematopoietic stem cell transplantation for autoimmune diseases: an observational study on 12 years’ experience from the European group for blood and marrow transplantation working party on autoimmune diseases. Haematologica. (2010) 95(2):284–92. doi: 10.3324/haematol.2009.013458 PMC2817032 19773265

[101] Tchao NK Turka LA. Lymphodepletion and homeostatic proliferation: Implications for transplantation: Lymphodepletion in transplantation. Am J Transplant (2012) 12(5):1079–90. doi: 10.1111/j.1600-6143.2012.04008.x 22420320

[102] Unsinger J Kazama H McDonough JS Hotchkiss RS Ferguson TA. Differential lymphopenia-induced homeostatic proliferation for CD4 + and CD8 + T cells following septic injury. J Leukocyte Biol (2009) 85(3):382–90. doi: 10.1189/jlb.0808491 PMC2653946 19088177

[103] Vuddamalay Y van Meerwijk JP. CD28. and CD28lowCD8+ regulatory T cells: Of mice and men. Front Immunol (2017) 8:31. doi: 10.3389/fimmu.2017.00031 28167946 PMC5256148

[104] Shevyrev D Tereshchenko V Manova O kozlov V. Research institute for fundamental and clinical immunology (RIFCI), novosibirsk, Russia, Samara state medical university, Samara, russia. homeostatic proliferation as a physiological process and a risk factor for autoimmune pathology. AIMS Allergy Immunol (2021) 5(1):18–32. doi: 10.3934/Allergy.2021002

[105] McKinney EF Lee JC Jayne DRW Lyons PA Smith KGC. T-Cell exhaustion, co-stimulation and clinical outcome in autoimmunity and infection. Nature. (2015) 523(7562):612–6. doi: 10.1038/nature14468 PMC4623162 26123020

[106] Thangavelu G Parkman JC Ewen CL Uwiera RRE Baldwin TA Anderson CC. Programmed death-1 is required for systemic self-tolerance in newly generated T cells during the establishment of immune homeostasis. J Autoimmunity (2011) 36(3–4):301–12. doi: 10.1016/j.jaut.2011.02.009 21441014

[107] Kasai M Nakagawa Y Kondo K Takahama Y. Thymus. In: Reference module in biomedical sciences. Tokushima, Japan:Elsevier, University of Tokushima (2014). doi: 10.1016/B978-0-12-801238-3.00109-4

[108] Serana F Chiarini M Zanotti C Sottini A Bertoli D Bosio A. Use of V(D)J recombination excision circles to identify t. and b-cell defects and to monitor the treatment in primary and acquired immunodeficiencies. J Transl Med (2013) 11(1):119. 23656963 10.1186/1479-5876-11-119 PMC3666889

[109] Levy A Rangel-Santos A Torres LC Silveira-Abreu G Agena F Carneiro-Sampaio M. T Cell receptor excision circles as a tool for evaluating thymic function in young children. Braz J Med Biol Res (2019) 52(7):e8292. 31241713 10.1590/1414-431X20198292 PMC6596370

[110] Hazenberg MD Verschuren MC Hamann D Miedema F Dongen JJ. T Cell receptor excision circles as markers for recent thymic emigrants: basic aspects, technical approach, and guidelines for interpretation. J Mol Med (2001) 79(11):631–40. 10.1007/s001090100271 11715066

[111] Alexander T Arnold R Hiepe F Radbruch A. Resetting the immune system with immunoablation ad autologous haematopoietic stem cell transplantation in autoimmune diseases. Clin Exp Rheumatol (2016) 34(4 Suppl 98):53–7. doi: 10.1586/ehm.09.60 27586805

[112] Raphael I Nalawade S Eagar TN Forsthuber TG. T Cell subsets and their signature cytokines in autoimmune and inflammatory diseases. Cytokine. (2015) 74(1):5–17. 25458968 10.1016/j.cyto.2014.09.011 PMC4416069

[113] Chizzolini C Brembilla NC Montanari E Truchetet M-E. Fibrosis and immune dysregulation in systemic sclerosis. Autoimmun Rev (2011) 10(5):276–81. 10.1016/j.autrev.2010.09.016 20863906

[114] Gasparini G Cozzani E Parodi A. Interleukin-4 and interleukin-13 as possible therapeutic targets in systemic sclerosis. Cytokine. (2020) 125:154799. 31400638 10.1016/j.cyto.2019.154799

[115] Argobi Y Smith GP. Fibrosis and immune dysregulation in systemic sclerosis. In: Willis MS Yates CC Schisler JC, editors. Fibrosis in disease. Cham: Springer International Publishing (2019). p. 25–60. Available at: http://link.springer.com/10.1007/978-3-319-98143-7_2.

[116] Meloni F Solari N Cavagna L Morosini M Montecucco CM Fietta AM. Frequency of Th1, Th2 and Th17 producing T lymphocytes in bronchoalveolar lavage of patients with systemic sclerosis. Clin Exp Rheumatol (2009) 27(5):765–72. 19917158

[117] Mavalia C Scaletti C Romagnani P Carossino AM Pignone A Emmi L. Type 2 helper T-cell predominance and high CD30 expression in systemic sclerosis. Am J Pathol (1997) 151(6):1751–8. PMC1858349 9403725

[118] Oliver SJ. The Th1/Th2 paradigm in the pathogenesis of scleroderma, and its modulation by thalidomide. Curr Rheumatol Rep (2000) 2(6):486–91. doi: 10.1007/s11926-000-0025-7 11123102

[119] Shah A Storek J Woolson R Pinckney A Keyes-Elstein L Wallace PK. Lymphocyte subset abnormalities in early severe scleroderma favor a Th2 phenotype and are not altered by prior immunosuppressive therapy. Rheumatology (2022) keac015. doi: 10.1093/rheumatology/keac015 35108379 PMC9536786

[120] Frantz C Auffray C Avouac J Allanore Y. Regulatory T cells in systemic sclerosis. Front Immunol (2018) 9:2356. doi: 10.3389/fimmu.2018.02356 30374354 PMC6196252

[121] Wing JB Tanaka A Sakaguchi S. Human FOXP3+ regulatory T cell heterogeneity and function in autoimmunity and cancer. Immunity. (2019) 50(2):302–16. doi: 10.1016/j.immuni.2019.01.020 30784578

[122] Antiga E Quaglino P Bellandi S Volpi W Del Bianco E Comessatti A. Regulatory T cells in the skin lesions and blood of patients with systemic sclerosis and morphoea: Regulatory T cells in scleroderma. Br J Dermatol (2010) 162(5):1056–63. doi: 10.1111/j.1365-2133.2010.09633.x 20105169

[123] Fenoglio D Battaglia F Parodi A Stringara S Negrini S Panico N. Alteration of Th17 and treg cell subpopulations co-exist in patients affected with systemic sclerosis. Clin Immunol (2011) 139(3):249–57. doi: 10.1016/j.clim.2011.01.013 21419712

[124] Papp G Horvath I Barath S Gyimesi E Sipka S Szodoray P. Altered T-cell and regulatory cell repertoire in patients with diffuse cutaneous systemic sclerosis. Scandinavian J Rheumatol (2011) 40(3):205–10. doi: 10.3109/03009742.2010.528021 21366383

[125] Scala E Pallotta S Frezzolini A Abeni D Barbieri C Sampogna F. Cytokine and chemokine levels in systemic sclerosis: relationship with cutaneous and internal organ involvement. Clin Exp Immunol (2004) 138(3):540–6. doi: 10.1111/j.1365-2249.2004.02642.x PMC1809238 15544634

[126] Schniering J Maurer B Distler O. Vascular mechanisms of systemic sclerosis. In: Matucci-Cerinic M Denton CP, editors. Atlas of ulcers in systemic sclerosis. Cham: Springer International Publishing (2019). p. 27–37.

[127] Hügle T O'Reilly S Simpson R Kraaij MD Bigley V Collin M. Tumor necrosis factor-costimulated T lymphocytes from patients with systemic sclerosis trigger collagen production in fibroblasts. Arthritis Rheumatol (2013) 65(2):481–91. doi: 10.1002/art.37738 PMC6588536 23045159

[128] Sato S Hasegawa M Fujimoto M Tedder TF Takehara K. Quantitative genetic variation in CD19 expression correlates with autoimmunity. J Immunol (2000) 165(11):6635–43. doi: 10.4049/jimmunol 11086109

[129] Mehra S Walker J Patterson K Fritzler MJ. Autoantibodies in systemic sclerosis. Autoimmun Rev (2013) 12:340–54. doi: 10.1016/j.autrev.2012.05.011 22743034

[130] Kayser C Fritzler MJ. Autoantibodies in systemic sclerosis: Unanswered questions. Front Immunol (2015) 6:167. doi: 10.3389/fimmu.2015.00167 25926833 PMC4397862

[131] Lafyatis R O’Hara C Feghali-Bostwick CA Matteson E. B cell infiltration in systemic sclerosis–associated interstitial lung disease. Arthritis Rheumatol (2007) 56(9):3167–8. doi: 10.1002/art.22847 17763433

[132] De Santis M Bosello SL Peluso G Pinnelli M Alivernini S Zizzo G. Bronchoalveolar lavage fluid and progression of scleroderma interstitial lung disease: Scleroderma interstitial lung disease. Clin Respir J (2012) 6(1):9–17. doi: 10.1111/j.1752-699X.2010.00228.x 21801327

[133] Bosello S Angelucci C Lama G Alivernini S Proietti G Tolusso B. Characterization of inflammatory cell infiltrate of scleroderma skin: B cells and skin score progression. Arthritis Res Ther (2018) 20(1):75. doi: 10.1186/s13075-018-1569-0 29669578 PMC5907298

[134] Melissaropoulos K Daoussis D. B cells in systemic sclerosis: from pathophysiology to treatment. Clin Rheumatol (2021) 40(7):2621–31. doi: 10.1007/s10067-021-05665-z 33745085

[135] François A Chatelus E Wachsmann D Sibilia J Bahram S Alsaleh G. B lymphocytes and b-cell activating factor promote collagen and profibrotic markers expression by dermal fibroblasts in systemic sclerosis. Arthritis Res Ther (2013) 15(5):R168. doi: 10.1186/ar4352 24289101 PMC3978899

[136] Sato S Fujimoto M Hasegawa M Takehara K. Altered blood b lymphocyte homeostasis in systemic sclerosis: Expanded naive b cells and diminished but activated memory b cells. Arthritis Rheumatism (2004) 50(6):1918–27. doi: 10.1002/art.20274 15188368

[137] Mavropoulos A Simopoulou T Varna A Liaskos C Katsiari CG Bogdanos DP. Breg cells are numerically decreased and functionally impaired in patients with systemic sclerosis: BREG CELLS IN SYSTEMIC SCLEROSIS. Arthritis Rheum (2016) 68:494–504. doi: 10.1002/art.39437 26414243

[138] Matsushita T Hamaguchi Y Hasegawa M Takehara K Fujimoto M. Decreased levels of regulatory b cells in patients with systemic sclerosis: association with autoantibody production and disease activity. Rheumatology (2016) 55:263–7. doi: 10.1093/rheumatology/kev331 26350483

[139] Aravena O Ferrier A Menon M Mauri C Aguillón JC Soto L. TIM-1 defines a human regulatory b cell population that is altered in frequency and function in systemic sclerosis patients. Arthritis Res Ther (2017) 19(1):8. doi: 10.1186/s13075-016-1213-9 28103916 PMC5248463

[140] Abrahamsson S Muraro PA. Immune re-education following autologous hematopoietic stem cell transplantation. Autoimmunity. (2008) 41(8):577–84. doi: 10.1080/08916930802197081 18958748

[141] Lutter L Spierings J van Rhijn-Brouwer FCC van Laar JM van Wijk F. Resetting the T cell compartment in autoimmune diseases with autologous hematopoietic stem cell transplantation: An update. Front Immunol (2018) 9:767(April). doi: 10.3389/fimmu.2018.00767 29731752 PMC5920130

[142] Sullivan KM Muraro P Tyndall A. Hematopoietic cell transplantation for autoimmune disease: Updates from Europe and the united states. Biol Blood Marrow Transplant (2010) 16(1):S48–56. doi: 10.1016/j.bbmt.2009.10.034 PMC3448948 19895895

[143] Wei C Anolik J Cappione A Zheng B Pugh-Bernard A Brooks J. A new population of cells lacking expression of CD27 represents a notable component of the b cell memory compartment in systemic lupus erythematosus. J Immunol (2007) 178(10):6624–33. doi: 10.4049/jimmunol.178.10.6624 17475894

[144] Simon D Balogh P Bognár A Kellermayer Z Engelmann P Németh P. Reduced non-switched memory b cell subsets cause imbalance in b cell repertoire in systemic sclerosis. Clin Exp Rheumatol (2016) 14):1–7. 27056741

[145] Delemarre EM Van Den Broek T Mijnheer G Meerding J Wehrens EJ Olek S. Autologous stem cell transplantation aids autoimmune patients by functional renewal and TCR diversification of regulatory T cells. Blood. (2016) 127(1):91–102. doi: 10.1182/blood-2015-06-649145 26480932

[146] de Paula A Sousa A Malmegrim KC Panepucci RA Brum DS Barreira AA Carlos Dos Santos A. Autologous haematopoietic stem cell transplantation reduces abnormalities in the expression of immune genes in multiple sclerosis. Clin Sci (Lond) (2015) 128(2):111–20. doi: 10.1042/CS20140095 25116724

[147] Blair PA Norena LY Flores-Borja F Rawlings DJ Isenberg DA Ehrenstein MR. CD19+CD24hiCD38hi b cells exhibit regulatory capacity in healthy individuals but are functionally impaired in systemic lupus erythematosus patients. Immunity (2010) 32:129–40. doi: 10.1016/j.immuni.2009.11.009 20079667

[148] Flores-Borja F Bosma A Ng D Reddy V Ehrenstein MR Isenberg DA. CD19+CD24hiCD38hi b cells maintain regulatory T cells while limiting TH1 and TH17 differentiation. Sci Transl Med (2013) 5:173ra23. doi: 10.1126/scitranslmed.3005407 23427243

[149] Iwata Y Matsushita T Horikawa M DiLillo DJ Yanaba K Venturi GM. Characterization of a rare IL-10-competent b-cell subset in humans that parallels mouse regulatory B10 cells. Blood (2011) 117:530–41. doi: 10.1182/blood-2010-07-294249 PMC3031478 20962324

[150] Avery DT Deenick EK Ma CS Suryani S Simpson N Chew GY. B cell-intrinsic signaling through IL-21 receptor and STAT3 is required for establishing long-lived antibody responses in humans. J Exp Med (2010) 207:155–71. doi: 10.1084/jem.20091706 PMC2812540 20048285

[151] Mion F Tonon S Toffoletto B Cesselli D Pucillo CE Vitale G. IL-10 production by b cells is differentially regulated by immune-mediated and infectious stimuli and requires p38 activation. Mol Immunol (2014) 62:266–76. doi: 10.1016/j.molimm.2014.05.018 24970737

[152] Kosmidis ML Dalakas MC. Practical considerations on the use of rituximab in autoimmune neurological disorders. Ther Adv Neurol Disord (2010) 3(2):93–105. doi: 10.1177/1756285609356135 21179602 PMC3002645

[153] Chamberlain N Massad C Oe T Cantaert T Herold KC Meffre E. Rituximab does not reset defective early b cell tolerance checkpoints. J Clin Invest (2016) 126:282–7. doi: 10.1172/JCI83840 PMC4701568 26642366

[154] Garcia-Montoya L Villota-Eraso C Yusof MYM Vital EM Emery P. Lessons for rituximab therapy in patients with rheumatoid arthritis. Lancet Rheumatol (2020) 2(8):e497–509. doi: 10.1016/S2665-9913(20)30033-3 38273611

[155] Jordan S Distler JH Maurer B Huscher D van Laar JM Allanore Y. Effects and safety of rituximab in systemic sclerosis: an analysis from the European scleroderma trial and research (EUSTAR) group. Ann Rheum Dis (2015) 74:1188–94. doi: 10.1136/annrheumdis-2013-204522 24442885

[156] Ebata S Yoshizaki A Oba K Kashiwabara K Ueda K Uemura Y. Safety and efficacy of rituximab in systemic sclerosis (DESIRES): a double-blind, investigator-initiated, randomised, placebo-controlled trial. Lancet Rheumatol (2021) 3:e489–97. doi: 10.1016/S2665-9913(21)00107-7 38279402

[157] Baker KP Edwards BM Main SH Choi GH Wager RE Halpern WG. Generation and characterization of LymphoStat-b, a human monoclonal antibody that antagonizes the bioactivities of b lymphocyte stimulator. Arthritis Rheum (2003) 48:3253–65. doi: 10.1002/art.11299 14613291

[158] Daoussis D Melissaropoulos K Sakellaropoulos G Antonopoulos I TE M Simopoulou T. A multicenter, open-label, comparative study of b-cell depletion therapy with rituximab for systemic sclerosis-associated interstitial lung disease. Semin Arthritis Rheum (2017) 46:625–31. doi: 10.1016/j.semarthrit.2016.10.003 27839742

[159] Gordon JK Martyanov V Franks JM Bernstein EJ Szymonifka J Magro C. Belimumab for the treatment of early diffuse systemic sclerosis: Results of a randomized, double-blind, placebo-controlled, pilot trial. Arthritis Rheumatol (2018) 70(2):308–16. doi: 10.1002/art.40358 PMC6590997 29073351

